# Predicting the Threat Status of Mosses Using Functional Traits

**DOI:** 10.3390/plants13152019

**Published:** 2024-07-23

**Authors:** Sinan Gürlek, Ana Claudia Araújo, Neil Brummitt

**Affiliations:** 1 Department of Life Sciences, Silwood Park Campus, Imperial College London, Ascot SL5 7PY, UK; 2 Natural History Museum, Cromwell Road, South Kensington, London SW7 5BD, UK; a.araujo@nhm.ac.uk (A.C.A.); n.brummitt@nhm.ac.uk (N.B.)

**Keywords:** conservation, extinction risk, ecological traits, modeling, reproductive traits, threatened bryophytes

## Abstract

Mosses are an early lineage of the plant kingdom, with around 13,000 species. Although an important part of biodiversity, providing crucial ecosystem services, many species are threatened with extinction. However, only circa 300 species have so far had their extinction risk evaluated globally for the IUCN Red List. Functional traits are known to help predict the extinction risk of species in other plant groups. In this study, a matrix of 15 functional traits was produced for 723 moss species from around the world to evaluate the potential of such predictability. Binary generalized linear models showed that monoicous species were more likely to be threatened than dioicous species, and the presence of a sporophyte (sexual reproduction), vegetative reproduction and an erect (straight) capsule instead of a pendent (immersed) one lowers the risk of species extinction. A longer capsule, seta and stem length, as well as broader substrate breadth, are indicative of species with a lower risk of extinction. The best-performing models fitted with few traits were able to predict extinction risks of species with good accuracy. These models applied to Data Deficient (DD) species proved how useful they may be to speed up the IUCN Red List assessment process while reducing the number of listed DD species, by selecting species most in need of a full, detailed assessment. Some traits tested in this study are a novelty in conservation research on mosses, opening new possibilities for future studies. The traits studied and the models presented here are a significant contribution to the knowledge of mosses at risk of extinction and will help to improve conservation efforts.

## 1. Introduction

We are facing what some call the sixth mass extinction event, and even seemingly insignificant or widespread groups of organisms are under threat [1] with lower biodiversity leading to reduced ecosystem functioning [2]. Mosses are a crucial part of this biodiversity, inconspicuously providing ecosystem services that benefit humans [3], such as fixing atmospheric nitrogen [4], storing water—particularly in peatlands—and creating a microclimate within their surroundings to provide a habitat for a diverse range of organisms [3]. Although evidence for climate-driven range shifts in mosses is scant, as climate change continues species migration rises, or worse leads to extinction. To prevent this conservation actions are necessary [5,6]. Before any actions can be taken to prevent moss extinctions, it is essential that we understand why some species are more threatened and likely to become extinct than related species are.

Mosses (division Bryophyta) are an early terrestrial lineage of plants, with approximately 13,000 species worldwide [7]. The gametophyte (haploid) is the dominant phase and is responsible for photosynthesis [7]. In contrast, the sporophyte (diploid) is short-lived and is only responsible for spore production. Albeit a dominant phase in other plant groups, in mosses the sporophyte cannot persist on its own without the gametophyte [7]. Understanding how these structures work is important for selecting the traits investigated here.

The sporophyte is composed of a capsule (the structure containing the spores) and a seta (the stalk-like structure holding the capsule away from its surroundings). Since the spore is the major dispersal unit of mosses, the sporophyte has a crucial role in moss life cycles [7]: a lack of sporophytes has previously been linked to rarity in mosses [8]. The other major dispersal unit of mosses is vegetative reproduction, which is achieved by specialized vegetative propagules (gemmae, bulbils, etc.) [7,9]. The protonema, the earliest stage of the gametophyte, is more resistant to some environmental stresses such as drought than mature gametophytes can be, and might also act as a dispersal tool [7,10].

Mosses are poikilohydric (lacking the ability to maintain and/or regulate their water content, except in the family Polytrichaceae which has internal conduction tissues) and their growth depends on water availability [7,11]. However, they can stay dormant during dry periods and restart photosynthetic activity after rehydration as a form of drought resistance. Mosses’ microhabitat requirements, such as substrate, are extremely important [3,12] and, to some extent, the macrohabitat, the region where they are found, is less so [3]. These adaptations have allowed mosses to flourish, with some species found only in specialized substrates such as animal dung, and others occupying rocks over a whole hemisphere.

Despite their physiology, ecology and economic value, many species of mosses are threatened with extinction. It has been estimated that one-fifth of all bryophyte species may be threatened [13], the major causes being loss of natural habitats and climate change [3,13]. Extinction risk is evaluated under the IUCN Red List, which sets criteria for applying Red List categories to species [14]. In Europe, where the moss flora is well studied and the IUCN Red List status of species is well known [3], conservation efforts have achieved positive results, emphasizing the importance of knowing the threatened status of species and showing that conservation action based on bryophyte status can save them [3]. Despite this, however, the current IUCN Red List only contains global assessments for just over 200 species of mosses [15], although in Europe c.1800 species have been assessed on a regional basis [15].

On the other hand, the functional traits of mosses have been studied for some time. Functional traits are characteristics of an organism that can indirectly impact its growth, reproduction and survival [16]. Many of them, especially those related to reproduction and ecophysiology, are suspected to explain the biogeography of mosses [9,17]. For example, since wind connectivity has been found to correlate with floristic similarities in bryophytes [18], functional traits facilitating wind dispersal, such as wider capsules and smaller spore size [19], can lead to larger ranges in mosses [9]. Functional traits related to the ecophysiology of mosses can explain why some species tolerate drier environments [11,20], such as the long and narrow leaves in the moss genus *Syntrichia* Brid. being more efficient at water and carbon uptake [21]. The combination of functional traits found in a species is thought to give insights into mosses’ biogeography, and more precisely their range size and/or habitat preferences [20,22,23].

Functional traits might also be correlated with categories of the IUCN Red List [24]. Range information is frequently used in Red List assessments [25] as one of five criteria (Criterion B) to evaluate extinction risk, and functional traits correlated with dispersal might therefore help predict Red List status. A recent study of mosses in Spain, for example, found a link between functional traits, such as habitat specialization and the absence of sexual reproduction, and threatened species [23]. In summary, there is broad agreement that functional traits help to explain threat status [24], and studying these traits in mosses can help predict the Red List status for non-assessed species to better protect them.

Previous studies looking at such traits lacked a global approach; Calleja and colleagues [23] considered only mosses of Spain, van Zuijlen and colleagues [17] considered only mosses of Europe, while other trait-biogeography studies have been restricted to species found on a specific type of substrate [22,26]. Therefore, the results they offer lack a global predictive approach and can only be considered for a specific bryological flora, not mosses in general. In addition to the reproduction- and ecophysiology-related traits identified by previous studies, new functional traits, such as substrate breadth, are also included here and can open new possibilities for future functional trait studies of mosses. This paper aims to use a functional trait dataset compiled for a global sample of mosses to evaluate the relation between such functional traits and their threat status, use this information to create a statistical model to predict extinction risk in mosses, and apply this to indicate the threat status of Data Deficient (DD) species. By understanding these trait/threat status relationships for a more globally representative sample of moss species than has been analyzed to date, we aim to understand why some mosses are more threatened than others and to predict whether or not they are threatened so that research and conservation efforts can be implemented, overcoming the lack of a predictive approach from previous studies.

## 2. Results

**Figure 1:**
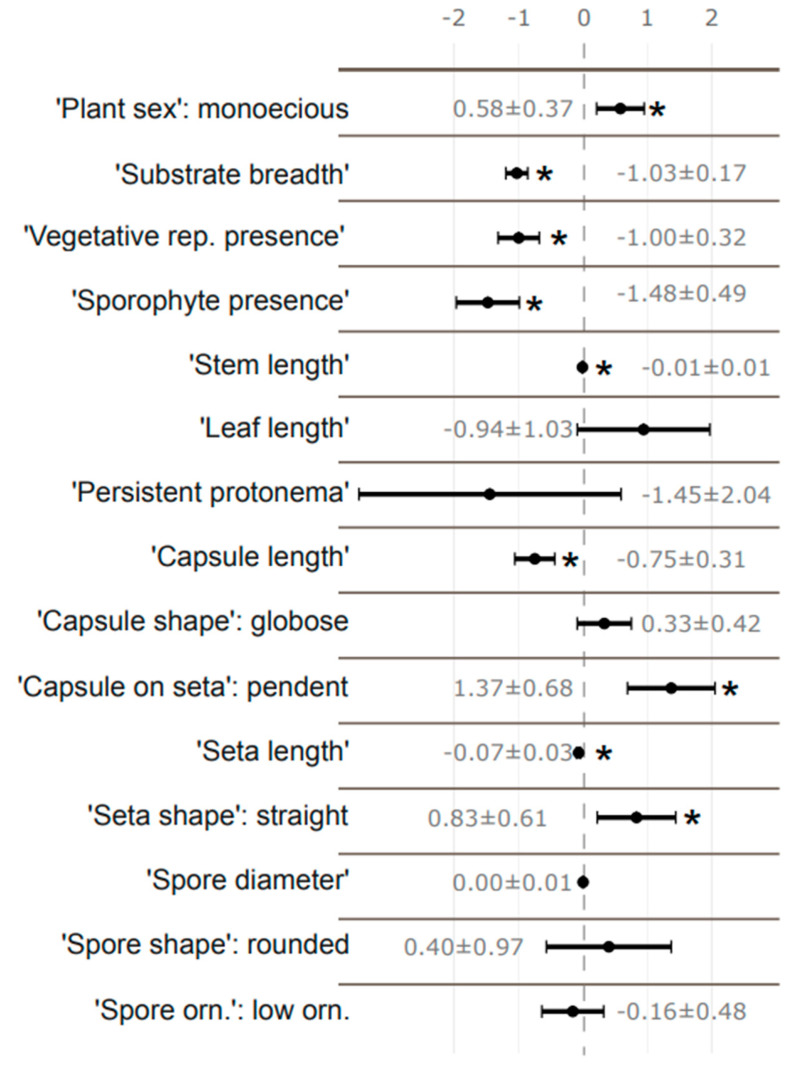
Binary GLM results. Estimate of coefficients (dots) with 95% confidence intervals (error bars) of the 15 functional traits, showing their respective numeric values beside the error bars. The significance is shown with asterisks (*) and can be visualized by the difference between the confidence intervals and intercept (vertical dashed 0 line).

Binary generalized linear models (GLMs) showed that out of fifteen traits evaluated (Figure 1), nine were significantly correlated with the binary threat status of mosses and had the potential to be applied as indicators to identify mosses under threat (represented in Figure 1 with an asterisk to show significance).

**Figure 2:**
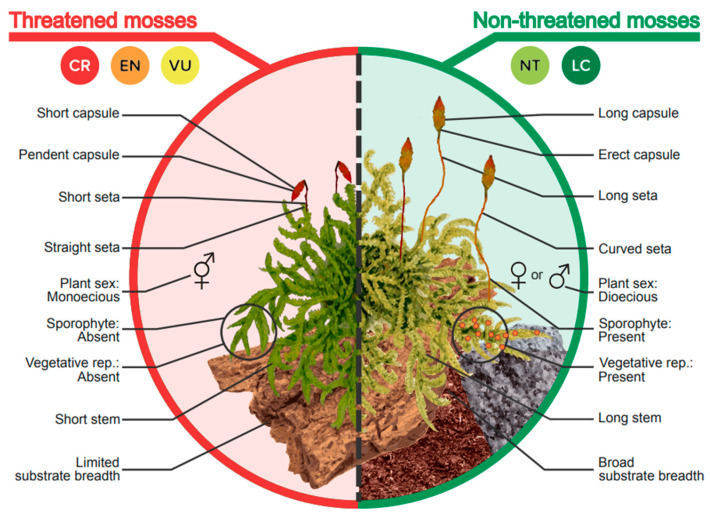
Functional traits with their attributes are shown on two illustrative moss plants, comparing these easy-to-see attributes that help indicate whether or not a species is more likely to be threatened.

Regarding reproduction-related traits, mosses that were monoicous (“Plant sex”), and/or lacked sporophytes (“Sporophyte presence”) and/or did not reproduce vegetatively (“Vegetative reproduction”) were more likely to be threatened than mosses that were dioicous, and/or bearing sporophytes and/or presenting vegetative reproduction (gemmae, bulbils, etc.). “Capsule length” and “Seta length” were also correlated with threat status, such that species with a shorter capsule and/or seta length were more likely to be threatened. Mosses with a “pendent” capsule (“Capsule on seta”) and a “straight seta” (“Seta shape”) were more likely to be threatened than mosses with an “erect” capsule and a “curved” seta (Figure 2). “Capsule shape”, “Spore diameter”, “Spore shape” and “Spore ornamentation” traits showed no significance and therefore were not useful in predicting the threat status of mosses.

Concerning ecophysiology-related traits, “Substrate breadth” (the number of different substrates a species can occupy) corresponded to a higher risk of a species being threatened when the range of substrates was limited. Likewise, the shorter the stem (“Stem length”), the higher the risk of a species being threatened (Figure 2). The “Leaf length” and “Persistent protonema” traits showed no significance and therefore are not useful for predicting the threat status of mosses.

Variance inflation factors ranged between 1 to 1.5, indicating little multicollinearity. A correlation between “Plant sex” and “Vegetative reproduction” was found (Pearson’s χ^2^(1) = 12.98; *p* ≤ 0.001): dioicous mosses more commonly reproduce vegetatively compared with monoicous mosses. Another correlation found was between “Plant sex” and “Sporophyte presence” (Pearson’s χ^2^(1) = 9.86; *p* = 0.002), with dioicous mosses more likely to lack a sporophyte than monoicous mosses. A final correlation was between “Seta length” and “Seta shape” (logit(‘Seta shape’)~log(‘Seta length’); Pearson’s χ^2^(1) = 4.33; *p* = 0.037), with increased logarithmic seta length values corresponding to a “curved” seta (coefficient = −0.19 ± 0.19, z-value = −2.03, *p*-value = 0.043). No further trait-to-trait correlation was found other than the ones mentioned above, and these correlations were taken into account during the model construction process.

From the results of the model selection process (Supplementary Material Table S3A,B), fitted models MAM1 and MAM2 showed good predictive capabilities (AUC of 0.71 and 0.75 respectively) for the threat status of mosses. MAM1 contained the traits “Plant sex”, “Sporophyte presence” and “Substrate breadth”, whereas MAM2 contained “Seta length” and “Substrate breadth” (Table 1 and Table 2). Equations for MAM1 and MAM2 are given below:

MAM1

$$Extinction\;Risk\;MAM1=\frac{e^{-0.79+0.54*X1-1.18*X2-0.83*\left(\frac{X3-1.69}{0.94}\right)}}{e^{-0.79+0.54*X1-1.18*X2-0.83*\left(\frac{X3-1.69}{0.94}\right)}+1}$$

X1: ‘plant sex’; 0 for dioicous and 1 for monoicous
X2: ‘sporophyte presence’; 0 for absence and 1 for presence
X3: ‘substrate breadth’; the number of different types of substrates occupied
Cutoff = 0.21 ≤ *Extinction Risk MAM1* ⇒ Species threatened
Cutoff = 0.21 > *Extinction Risk MAM1* ⇒ Species non-threatened

MAM2

$$Extinction\;Risk\;MAM2=\frac{e^{-1.65-0.61*(\frac{X1-14}{13.32})-0.9*\left(\frac{X2-1.8}{0.98}\right)}}{e^{-1.65-0.61*(\frac{X1-14}{13.32})-0.9*\left(\frac{X2-1.8}{0.98}\right)}+1}$$

X1: ‘seta length’; the length of seta in mm
X2: ‘substrate breadth’; the number of different types of substrates occupied
Cutoff = 0.18 ≤ *Extinction Risk MAM2* ⇒ Species threatened
Cutoff = 0.18 > *Extinction Risk MAM2* ⇒ Species non-threatened

According to MAM1 (Table 1), dioicous mosses without a sporophyte had a probability of 31% (21–44% with 95% CI) of being threatened, lower than monoicous mosses without a sporophyte which had a 44% probability (15–55% with 95% CI). Sporophyte presence lowered this probability to 12% for dioicous mosses (7–19% with 95% CI) and 19% for monoicous mosses (11–30% with 95% CI). An increase in “Substrate breadth” of one standard deviation (0.94, close to occupying one more substrate) resulted in the probability of being threatened decreasing 1.9-fold for dioicous mosses without a sporophyte. The cutoff to decide whether a moss is threatened or non-threatened was found to be 0.21.

According to MAM2 (Table 2), the baseline probability of a moss being threatened (for a “Seta length” of 14 mm and “Substrate breadth” of 1.8) was 17% (13–20% with 95% CI). An increase in “seta length” of one standard deviation (13.3 mm) resulted in this probability decreasing by 1.7-fold. One standard deviation increase in “substrate breadth” (0.98) resulted in this probability decreasing by 2.2-fold. The cutoff to decide whether a moss is threatened or non-threatened was found to be 0.18.

The Index of Union method found the optimal cutoff value to be 0.3 for MAM1 (for a sensitivity of 0.53 and a specificity of 0.74 with an AUC of 0.71) and 0.18 for MAM2 (for a sensitivity of 0.75 and a specificity of 0.62 with an AUC of 0.75). For MAM1, this optimal cutoff value corresponded to a lower sensitivity than expected (ideally above 0.7), so this optimal cutoff was not accepted. Instead, the cutoff value was taken to be 0.21, as this approximated to the third-best optimal cutoff value after the Index of Union method, where the sensitivity was high enough (0.75 with a specificity of 0.51). These cutoff values and the relative specificity/sensitivity values can be found in Supplementary Materials Figure S1.

**Figure 3:**
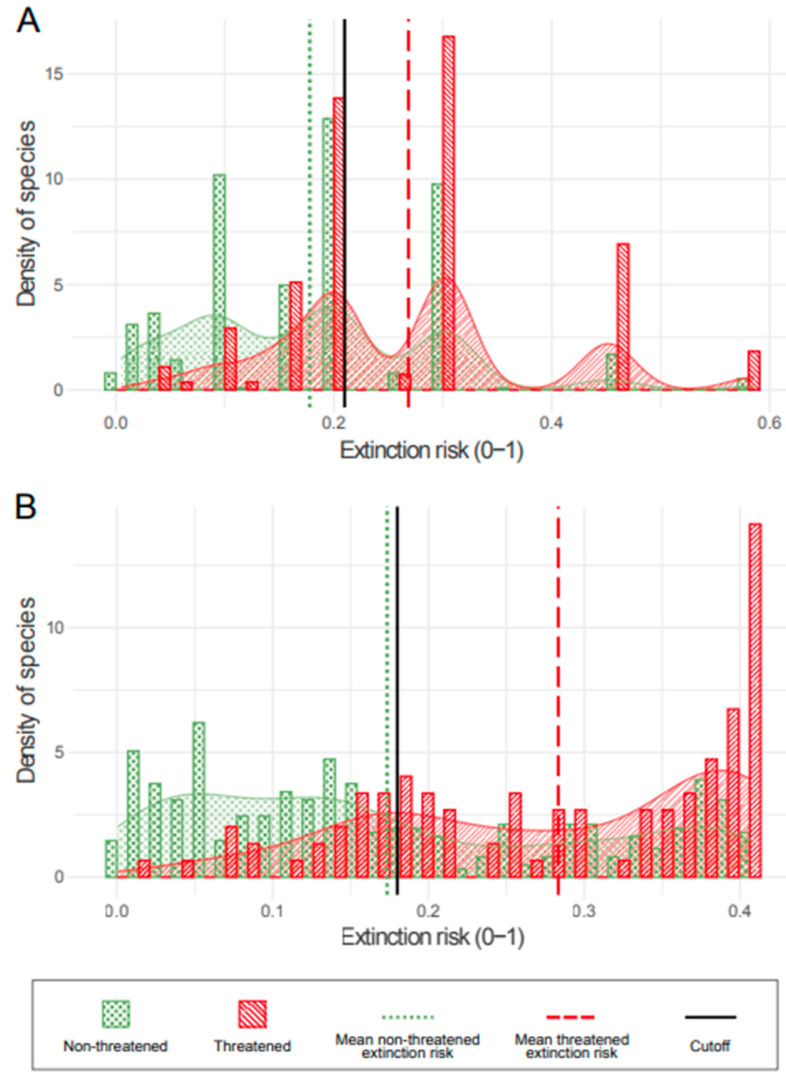
Extinction risks calculated by MAM1 (**A**) and MAM2 (**B**) for both threatened (red stripes) and non-threatened (green dots) species, with density of species on the y-axis and extinction risk on the x-axis. Mean extinction risk for non-threatened species (dotted green lines) and threatened species (dashed red lines) and cutoff values (solid black lines) for threatened/non-threatened species are shown.

With extinction risks for each species calculated by either only MAM1 or also MAM2, depending on the presence of sporophyte traits, it is possible to see how the threatened and non-threatened mosses were distinguished and the relative positions of the cutoffs among them (Figure 3). These results show that the extinction risks overlap for threatened and non-threatened species. The proximity of the cutoff values and the mean extinction risk for non-threatened species showcases this and is the product of the high sensitivities (75%) and low specificities (51% for MAM1 and 62% for MAM2) of the models.

**Figure 4:**
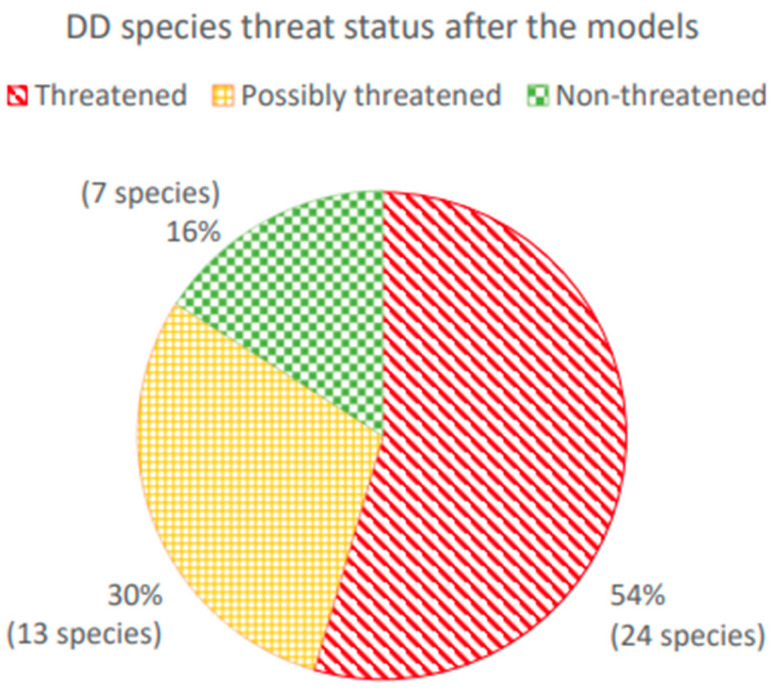
The threat status attributed to DD species after extinction risks were calculated by models MAM1 and MAM2, shown as “threatened” (when both possible models show the “threatened” result), “possibly threatened” (when one of the two models show the “threatened” result) and “non-threatened” (when both possible models show the “non-threatened” result).

Extinction risks were calculated and re-evaluated using the formulas in Table 1 and Table 2 for 44 species currently assessed as DD (Table 3). Figure 4 shows that 54% of currently classified DD species are predicted as being “threatened” according to both models, MAM1 and MAM2, or to only a single model when that is the only model available (for example, a species where attributes of the sporophyte are known has been modeled under MAM2); 30% of them are predicted to be threatened only by one model, when both models were tested (referred as “possibly threatened”) and 16% of DD species were found to be non-threatened (“non-threatened”). These results mean that the majority of mosses classified as DD are heading towards extinction, but also show that only 30% of them where the model results are not definite either way should remain as DD.

Table 3 lists 44 names assigned as DD at the time of investigation, from which 24 (54%), are predicted to be threatened (Thr.) based on their functional traits. From the remaining 20 names classified as DD, 13 (30%) reach the threshold to be possibly threatened (Poss. Thr.) while only seven species (16%) are predicted not to be threatened based on their functional traits. However, subsequent to their initial assessment as DD, taxonomic changes have been proposed for seven names (Table 3, “Accepted name”) where the rating has since also changed (Table 3, “Updated Rating”). While the updated ratings for *Acaulon triquetrum* agree with the results obtained in this study, those of *Andreaea rupestris*, *Coscinodon cribrosus* and *Phascum cuspidatum* do not. On the other hand, *Hyophila involuta*, found in this study to be “Possibly Threatened” when evaluated by its functional traits, has been assessed regionally as being Vulnerable.

## 3. Material and Methods

### 3.1. Data Collection

A total of 723 species were obtained from those assessed under IUCN Red List criteria, either for the IUCN Red List website [15] or for the Plants Under Pressure (PUP) research program at the Natural History Museum [13,27] (a full list is given in Supplementary Material Table S1), including Data Deficient (DD) species, resulting in a global list. Due to the low number of global IUCN Red List assessments for mosses, regional assessments were also considered, given that any species assessed regionally as Least Concern (LC) will also be LC globally under IUCN Criterion B; the full range of each regional LC species was also checked to confirm that this extended beyond an EOO of 20,000 km^2^. Species deemed “threatened” regionally were investigated further regarding their up-to-date distribution [28] and range information [15] to identify endemic species where a regional assessment equates to a global one. The categories considered to be “threatened” (162 species) were Extinct (EX), Critically Endangered (CR), Endangered (EN), and Vulnerable (VU), while “non-threatened” (567 species) included species assessed as being Near-Threatened (NT) or Least Concern (LC).

Multiple reproduction and ecophysiology-related functional traits were evaluated. The data were obtained by applying Natural Language Processing (NLP) machine learning (ML) algorithms, developed together by the Plants Under Pressure and Informatics teams of the Natural History Museum, London (Scott et al., in prep.), which automatically extract species trait information from online taxonomic descriptions of species or higher taxa. Online versions of the Flora of North America [29], Flora of China [30] and the vast corpus available via the Biodiversity Heritage Library [31] were utilized. Gaps in the data acquired automatically by ML were then filled manually by checking these sources, the World Flora Online [32] and other specialized literature. Where possible, remaining missing data (traits not automatically or manually found in species descriptions) were sourced from taxonomic descriptions at the genus/family level and from viewing online specimen images provided by online sources such as the Global Plants Initiative [33] and Global Biodiversity Information Facility [28], including under the species’ accepted name and synonyms/basionyms where available. Presence of a sporophyte, the length and shape of seta and capsule, and position of capsule may be observed from specimen images. Some functional traits considered initially were later discarded from analyses due to inconsistencies in their recording in species’ taxonomic descriptions.

Fifteen functional traits were investigated, after eliminating traits where attributes were either applied interchangeably between two distinct forms or were reported inconsistently in the taxonomic descriptions (see Supplementary Material Table S2A–C for summary statistics). For categorical functional traits, attributes were grouped further (Supplementary Material Table S2A). In the case of continuous functional traits (Supplementary Material Table S2B), mean values were used; data were scaled to z-scores before statistical analysis. “Substrate breadth”, a new functional trait, was calculated as the sum of different substrates a species has been recorded as occupying; equal weighting was used for different substrates. For binary-valued traits (Supplementary Material Table S2C), where only the presence of certain structures is reported in the taxonomic descriptions (e.g., “vegetative reproduction present”), empty cells in the data were considered to be the absence of that particular structure.

### 3.2. Data Analysis

Threat status (0 for non-threatened and 1 for threatened species) and individual functional traits were modeled using a generalized linear model (GLM) with a logistic link function (logit(threat status)~functional trait). Functional traits that significantly correlated with threat status were then selected to create maximal models using the same GLM approach. To avoid multicollinearity, correlations between functional traits were checked using the “vif” (variance inflation factor) function before fitting the maximal models, which were also used to generate possible explanations for the significant functional traits. Since some mosses are not known to produce sporophytes (i.e., taxonomic descriptions stating the absence of the sporophyte) or had not produced them at the moment of observation (i.e., taxonomic descriptions stating the presence of the sporophyte but not mentioning capsule, seta or spore traits), two maximal models were fitted: one model containing only the traits that are not sporophyte-related as well as the binary trait “Sporophyte presence”, “yes” or “no” (maximal model 1), and the other model with all the possible traits except “Sporophyte presence” (maximal model 2). In this way it was possible to model the extinction risk for every species of moss, irrespective of whether sporophyte traits are known or not; those mosses known to possess sporophytes, where attributes of sporophyte traits are recorded, were also modeled separately using the alternative model. Significant functional traits were added successively with automated stepwise forward model selection using the AIC (Akaike Information Criterion) (the stepCriterion function from the “glmtoolbox” package [34]). Minimum adequate models (MAM1 and MAM2 for maximal models 1 and 2, respectively) were selected from the lowest AIC scores. Binned residual plots were used to confirm the GLM assumptions. 

The cutoff for a species to be considered threatened was determined using the Index of Union method [35] and was visualized using two-graph receiver operator characteristic curves [36] for both MAM1 and MAM2. Since the aim of these models is to prioritize the detection of truly threatened species (true positives) rather than to minimize the misidentification of non-threatened species (false positives), sensitivity was prioritized over specificity. Therefore, cutoff values where the sensitivity was higher compared to the specificity were chosen, even if the Index of Union method failed to do this. Extinction risks for DD species were calculated using the MAM1 and MAM2 model parameters and were assigned to be threatened or non-threatened using the cutoff values. Every DD species was modeled using MAM1; where sporophyte traits were recorded, those DD species were also modeled using MAM2.

The analysis was performed in R version 4.3.1 [37]. Packages “dplyr” [38], “stringr” [39] and “tidyr” [40] were used for data wrangling, “regclass” [41], “pROC” [42] and “AICcmodavg” [43] for additional model selection parameters, “readxl” [44] for trait data importing, and “ggplot2” for plots [45].

## 4. Discussion

The nine functional traits that were significantly correlated with mosses’ extinction risk were either reproduction- or ecophysiology-related. Starting with reproduction-related traits, most dioicous species were less threatened when compared with monoicous species, similar to results found in previous studies [8,17,23,46]. The advantage of dioecy could be linked to the high dimorphism between female and male plants of certain species [47,48], perhaps giving increased fitness to some dioicous mosses [49], although testing adaptive fitness per se was not the objective of the analyses. Another advantage of dioecy could be a higher vegetative reproduction rate in dioicous mosses, as discovered during this investigation, which might explain why dioicous species seem to cope well with the absence of sporophytes [50]. In contrast, dioecy in mosses can result in sporophytes being formed rarely, which is disadvantageous for moss dispersal, as discussed below and in agreement with other studies [8,48]. Dioicous mosses lack a sporophyte more often than monoicous mosses do; on the other hand, the trait “Sporophyte presence” may not be sufficient to study the phenomenon of rare sporophyte formation in dioicous mosses, since in this study the trait only represents the presence or absence of the sporophyte and not its frequency of occurrence.

The presence of sporophytes corresponded to a lower risk of extinction, showing the advantages of sexual reproduction and the applicability of reproduction-related traits to predict extinction risk in mosses. This result was expected since sexual reproduction seems to be associated with reduced rarity [8] and results in genetic variability within populations, producing fitter colonies [48]. It results in the production of spores, the major long-distance dispersal units of mosses [7]. Vegetative reproduction seems to be more common in dioicous mosses [8,23], allowing organisms to increase their colony size rapidly and compete with other species successfully [7,23], perhaps explaining why it was significantly related to threat status and why its correlation with “Plant sex” can be advantageous for dioicous mosses: the frequency of vegetative reproduction may compensate for sporophyte absence in dioicous mosses, explaining why dioicous species remain less likely to become threatened, even when lacking a sporophyte.

“Capsule length” was also investigated, as one can expect longer capsules to be larger and large capsules to produce more spores. A higher spore number increases the chances of a successful long-distance dispersal event [51], explaining the correlation between “Capsule length” and lower extinction risk. In addition to its length, the orientation of the capsule on top of the seta (“Capsule on seta”) also proved to be important, probably due to the spore release phenomenon [52]: a capsule with its opening facing toward the substrate (a “pendent” capsule) will have a lower spore-release threshold and thus shed its spores at relatively low wind speeds when the chances of long-distance dispersal are lower [53]. In contrast, a capsule with an opening facing outwards (an “erect” capsule) should be more likely to disperse its spores at higher wind speeds (increased spore-release threshold), when they can be carried long distances [53]. In most of the reproduction-related traits such as “Capsule on seta”, attributes promoting long-distance dispersal events were more likely to be found in non-threatened mosses compared with immediate, short-distance dispersal events, showing the importance of long-distance dispersal for lowering extinction risk by increasing species range size [54], as evaluated under Criterion B in the IUCN Red List Categories and Criteria [14].

The seta plays a fundamental role in releasing spores [7,52] and its presence increases successful dispersal. A short seta (“Seta length”) may limit long-distance spore dispersal [52] therefore compromising range size and increasing extinction risk [54]. Although “seta shape” was also found to affect the threat status of mosses, this is likely to be a product of the correlation with seta length, as a longer seta bends more easily under the weight of the capsule and thus becomes curved. For that reason, “seta shape” was not used in the model selection process. However, it is a good visual clue for field studies, where “seta shape” alone can be a sign of seta length, without the need to use the model parameters.

Regarding ecophysiology-related traits, “Substrate breadth” was investigated for the first time in this study. It is a refinement of the well-studied “habitat breadth” trait, which looks at the occupation of different habitats by a species and is negatively correlated with extinction risk in many terrestrial taxa [55]. As microhabitats such as substrate are important for mosses [3,12], the habitat was substituted with substrate information in this trait. Results show that moss species that can occupy multiple substrates are less likely to be threatened. The lack of weighting for different substrates means that here the abundance and suitability of two different substrates are treated as the same. A species growing only on one single but abundant substrate (e.g., *Orthotrichum casasianum* growing only on tree bark [56] should perhaps not have the same “Substrate breadth” value as a species that grows only on a rare substrate (e.g., *Splachnum luteum*, which grows only on large herbivore dung [29]. Nevertheless, as attributing a non-arbitrary weighting for these substrates was not possible without a specific investigation of this trait alone, in this study all types of substrates were considered as separate microhabitats for mosses. The statistical significance of this trait, even without any weighting, shows how strongly it is correlated with extinction risk, opening a new avenue for future moss studies.

“Stem length”, a second ecophysiology-related trait approximating plant height for mosses, was significantly correlated to threat status, where longer stems correspond to decreased extinction risk. Another recent study by Van Zuijlen and colleagues [17] found this exact correlation, showing the need for a careful study of moss stem length. Other than this recent publication, which provides strong arguments on why this might be the case, the reasoning behind the significance of stem length seems to be lacking in the literature. It is known that for small vertebrates habitat destruction and pollution might have more adverse effects [57] but no similar study has been conducted on relative plant size. It is worth mentioning that a recent study [55] failed to find such a size (referred to as “plant height” in that study) and threat status correlation in bryophytes.

Both reproduction- and ecophysiology-related traits were correlated with the threat status of mosses. Since MAM1 and MAM2 models were constructed from these traits, they are easily applicable and, based on the AUC scores, accurate enough to predict the threat status of mosses. The MAM1 and MAM2 formulae provided can be used quickly to calculate the extinction risk of mosses without an IUCN Red List assessment but where the taxonomic description is available, and decide whether or not they are more likely to be threatened. Where possible, the two models should be used simultaneously for this decision, as they use different functional traits that might explain different threat aspects of the mosses. Needless to say, MAM2 cannot be used for extinction risk calculations in moss species lacking sporophytes, as the model is only for sporophyte-bearing species. The extinction risk calculated by these models is a continuous variable, so an appropriate cutoff therefore had to be determined to attribute a binary threat status to mosses. As the models contained two or three traits, the extinction risk results for threat status overlapped between threatened and non-threatened mosses (Figure 3), making it hard to determine a perfect cutoff that separated species with precision by their extinction risk.

Considering the detailed assessment criteria of the IUCN Red List [14], it is impossible to avoid these overlapping areas of extinction risk when only a few functional traits are available. Note that since some phylogenetically close groups have the same functional trait attributes for the traits concerning the GLM models presented here, it is inevitable that they will score the same extinction risks. This is the outcome of using a low-specificity model. However, this should not mean inaccuracy of the models in determining threatened species. Since the aim of these models was primarily to identify threatened species rather than non-threatened species, high specificity was chosen, with a trade-off of more false positives (falsely threatened) but fewer false negatives (falsely non-threatened). Species indicated to be threatened by these models should still be evaluated carefully by applying the IUCN Red List Categories and Criteria as they are likely to be threatened by extinction if not previously assessed. Species already assessed as Data Deficient (DD) require further investigation by specialists to evaluate their taxonomic status as well as their threat status.

To showcase this, a number of DD species were gathered and analyzed. With the two-model strategy, it was possible to model the extinction risk for species without sporophytes using MAM1, as this does not contain any sporophyte-related traits other than the “Sporophyte presence” trait. For species with sporophytes, both MAM1 and MAM2 models were used, increasing the number of functional traits to predict extinction risk and thus giving deeper insights into trait/threat relationships of mosses. From the results of the models, for any species (and not the DD species only), one of these three facts can be said. First, if both models give a non-threatened result, a species should be considered as non-threatened in the absence of a full IUCN Red List assessment (16% of the assessed DD species presented here). Second, the models may give contradictory results (species classified here as “possibly threatened”, 30% of the DD species included here). Treating these species as “possibly threatened” applies a precautionary principle and a full IUCN Red List assessment should be undertaken. The third and last group has both models indicating that a species is likely to be threatened (54% of the assessed DD species presented here), and these species can be prioritized for a full IUCN Red List assessment to understand the exact causes behind their risk of extinction. With 54% of these DD species found to be threatened according to these models and a further 30% to be possibly threatened, it is crucial to highlight the extinction risk faced by the majority of mosses currently within the DD category, to clarify the taxonomic status of these species, and to recognize the importance of undertaking full IUCN Red List assessments to ensure the success of any resulting conservation efforts.

However, since the models have low specificities, it is possible that some of the DD species here predicted to be threatened are not currently threatened by extinction. Although some of the traits analyzed here are associated with species’ range size, threats are usually specific and localized, so range and threats can interact in different ways: species may have a small range and be predicted to be threatened (as most of the DD species here are—they have every justification to be considered threatened under IUCN Red List Criteria, as they are unlikely to reproduce sexually or disperse successfully); may have a small range and be predicted as not being threatened (these naturally rare species should continue to be monitored and might perhaps be considered equivalent to Near Threatened species in the IUCN Red List); may not be predicted as being threatened and have a large range, as most LC species do; or indeed may be rated as LC due to a large range but still be predicted to be threatened. By combining the observed functional traits and range size in this way, the problem of low specificity of the models can be surpassed, discarding many falsely threatened species and prioritizing assessments of moss species that show a greater likelihood of truly being or becoming threatened.

There are many reasons to classify a species as DD, one of them being ongoing taxonomic uncertainty [14]. As observed in Table 3, seven names initially evaluated as accepted DD species have since been found to be synonyms of other species. These changes mean that some of these initial DD ratings now reflect species with a wider range, resulting in an apparent contradictory threat status when compared to the models’ findings. For instance, *Andreaea rupestris*, *Coscinodon cribrosus* and *Phascum cuspidatum* (Table 3, under “Accepted name”) are classified as LC [15], whereas the analysis presented here indicates that these taxa are threatened, under their former names. It is important to consider that the analysis presented here can evaluate the capacity of a species to endure threats successfully, based on its functional traits, particularly when faced with habitat loss. Likewise, this set of analyses is proposed in order to prioritize species alongside a full evaluation of their extinction risk. Nonetheless, these results highlight the underlying reproductive or eco-physiological trait attributes of threatened mosses.

In this respect, another important point to highlight is that for most plant species currently classified as LC, this is due to their wide range, which is usually evaluated based on herbarium records. Some of these may be hundreds of years old, but areas where they no longer exist due to recent habitat loss are not always excluded. Those species that have a wide range but even so have the trait attributes that predict them as being threatened are considered the most important group of mosses identified here since their range size generally means that they would be automatically rated as being non-threatened under IUCN Red List criteria. However, because of their trait attributes, these mosses might still become threatened as a result of decreasing range in the future, due to their low capacity for reproduction and dispersal. Experts should consider monitoring and re-evaluating these species regularly for loss of local populations, alongside evaluation of the ecosystem that supports them. Many LC species have suffered habitat reduction subsequently and no longer occur where they were once recorded; this seems to be the case with *Hyophila involuta*, which, although it has a very wide range [28], has been assessed regionally as threatened [15].

Ground truthing and a thorough re-evaluation of the species every 10 years are fundamental to revealing genuine changes in Red List status. However, ground truthing is an expensive and time-consuming process, while re-assessments of species may be lengthy and do not necessarily improve knowledge. Therefore, a species classified as threatened by these models may be truly threatened now or could be susceptible to becoming threatened in the future by habitat loss or climate change. These models will thus help experts to prioritize species that have no IUCN Red List assessment, as well as contribute to monitoring and re-evaluation efforts of species already prioritized due to loss of local populations, as well as their local ecosystem.

## 5. Conclusions

In this study, seven reproduction- and two ecophysiology-related functional traits were found to correlate with moss species at higher risk of extinction. These nine traits were “Plant sex”, “Sporophyte presence”, “Vegetative reproduction”, “Capsule length”, “Capsule on seta”, “Seta length”, “Seta shape”, “Substrate breadth” and “Stem length”. “Substrate breadth” is a novelty for the literature, opening new possibilities for future studies. Models constructed from these traits had good predictive capabilities, enabling mosses most likely to be at risk of extinction to be predicted rapidly, based on trait information readily available in taxonomic literature, which reflects their abilities of surviving these threats. The simplicity and the novelty of these functional traits, together with the accuracy of the models, open the opportunity to efficiently identify moss species at higher risk of extinction before they become threatened. These predictions, in turn, though not as detailed as IUCN assessments, can highlight those species that should be prioritized for rigorous assessments using the IUCN Red List Categories and Criteria. The 54% share of DD moss species found to be threatened using these models is a striking example of how many species need our attention before facing extinction. We hope that, in turn, this contribution will speed up the assessment process to identify truly threatened species and more successfully support conservation initiatives that will change what otherwise would be inevitable.

## Figures and Tables

**Table 1:** GLM results for MAM1, showing estimates with 95% confidence intervals (CI), z-values and *p*-values, in addition to the full equation for finding the extinction risk. The cutoff value is given as well as its relation to the threat status.

Coefficients	MAM1 Estimate ± CI (95%)	z-Value	*p*-Value
Intercept: Dioicous	−0.79 ± 0.54	−2.85	0.004
Plant sex: Monoicous	0.54 ± 0.40	2.62	0.009
Sporophyte presence	−1.18 ± 0.57	−4.05	<0.001
Substrate breadth	−0.83 ± 0.32	−5.00	<0.001

df = 696; N = 700 (137 threatened, 563 non-threatened); AUC = 0.71.

**Table 2:** GLM results for MAM2, showing estimates with 95% confidence intervals (CI), z-values and *p*-values, in addition to the full equation for finding the extinction risk. The cutoff value is given as well as its relation to the threat status.

Coefficients	MAM2 Estimate ± CI (95%)	z-Value	*p*-Value
Intercept	−1.65 ± 0.61	−11.99	<0.001
Seta length	−0.61 ± 0.58	−3.58	<0.001
Substrate breadth	−0.90 ± 0.79	−4.90	<0.001

df = 541; N = 544 (106 threatened, 438 non-threatened); AUC = 0.75.

**Table 3:** DD (Data Deficient) species of bryophytes modeled for this paper to determine whether or not their IUCN Red List status might potentially be threatened. Accepted names (**bold**), synonyms (*italic*). “Species” is the name under which the taxon was originally assessed by IUCN (“globally” indicated by *, “regionally” indicated by ^‡^) or by PUP (without symbol); “Accepted name” gives the currently accepted taxonomic name, where this has since been updated, together with their respective “Updated rating” evaluated by IUCN [15], either global (*) or regional (^‡^). “MAM1” and “MAM2” bootstraps indicate predicted extinction risk. “Verdict”: Non-Thr. (non-threatened), Poss. Thr. (possibly threatened), Thr. (threatened).

Taxon	MAM1	MAM2	Modeled Rating	Accepted Name	Updated IUCN Rating
*Acaulon piligerum* (De Not.) Limpr. *	0.069	0.107	Non-Thr.	**Acaulon triquetrum** (Spruce) Müll. Hal.	LC *
**Acroporium consanguineum** (Broth.) M. Fleisch.	0.304	0.417	Thr.		
**Aloina humilis** M.T. Gallego, M.J. Cano & Ros *	0.304	0.368	Thr.		
*Andreaea alpestris* (Thed.) Schimp. *	0.304	0.413	Thr.	**Andreaea rupestris** Hedw.	LC *
**Andreaea vaginalis** Herzog	0.203		Non-Thr.		
**Barbula lavardei** (Thér.) R.H. Zander & S.P. Churchill *	0.453		Thr.		
**Barbula macassarensis** M. Fleisch.	0.203		Non-Thr.		
**Barbula novogranatensis** Hampe *	0.203	0.373	Poss. Thr.		
**Barbula stenocarpa** Hampe *	0.304	0.356	Thr.		
**Bartramia aprica** Müll. Hal. *	0.304	0.313	Thr.		LC ^‡^
**Brachymenium curvitheca** Dixon	0.203	0.072	Non-Thr.		
**Braunia schimperi** Bruch & Schimp.	0.304	0.313	Thr.		
**Bryum demaretianum** Arts *	0.255		Thr.		
**Bryum enisseense** L.I. Savicz	0.304	0.232	Thr.		
**Callicostella mosenii** (Broth.) Broth.		0.200	Thr.		
**Chionoloma minus** (Köckinger, O. Werner & Ros) M. Alonso, M.J. Cano & J.A. Jiménez *	0.255		Thr.		
**Cinclidotus x vivesii** Ederra	0.453		Thr.		
*Coscinodon humilis* Milde *	0.203	0.411	Poss. Thr.	**Coscinodon cribrosus** (Hedw.) Spruce	LC *
**Didymodon soaresii** Luisier	0.203		Non-Thr.		
**Distichophyllum telmaphila** Colenso		0.297	Thr.		
**Drepanocladus halli** Broth. & Dixon *	0.203		Non-Thr.		
**Drepanocladus sparsus** *		0.274	Thr.		
**Entosthodon clavatus** Müll. Hal.	0.304	0.363	Thr.		
**Grimmia arenaria** Hampe *	0.203	0.407	Poss. Thr.		
**Grimmia laevigata** (Brid.) Brid. *	0.203	0.405	Poss. Thr.		LC ^‡^
**Helicoblepharum daltoniaceum** (Hampe) Broth. *	0.203	0.271	Poss. Thr.		
*Hyophila bingeri* Broth. & Paris ^‡^	0.203	0.419	Poss. Thr.	**Hyophila involuta** (Hook.) A. Jaeger	VU *
*Hyophila latifolia* Broth. ^‡^	0.203	0.357	Poss. Thr.	**Hyophila involuta** (Hook.) A. Jaeger	VU *
**Hypnella punctata** Broth.	0.453		Thr.		
*Hypnum aemulans* Breidl. *	0.453		Thr.	**Stereodon aemulans** (Breidl.) Broth.	DD *
**Isopterygium plumicaule** (Müll. Hal.) W.R. Buck	0.304	0.380	Thr.		
**Lepidopilidium cespitosum** (Besch.) Broth.	0.203	0.201	Poss. Thr.		
**Orthotrichum cambrense** Bosanquet & F. Lara *	0.304	0.420	Thr.		
**Philonotis striatula** (Mitt.) A. Jaeger *	0.304	0.031	Poss. Thr.		
**Pseudoleskea dispersa** Müll. Hal.	0.453		Thr.		
**Pterygoneurum papillosum** Oesau *	0.153	0.208	Poss. Thr.		
**Pterygophyllum chonoticum** Mitt.		0.198	Thr.		
**Racopilum crassicuspidatum** Thér. & Corb.		0.230	Thr.		
**Rhynchostegiella tubulosa** Hedenäs & J. Patiño *	0.304		Thr.		
**Sematophyllum fragilirostrum** (Hampe) Mitt. *	0.203	0.273	Poss. Thr.		
**Taxithelium ramivagum** Broth.	0.203	0.323	Poss. Thr.		
**Trematodon brevifolius** Broth. ex Müll. Hal.	0.304	0.338	Thr.		
*Weissia multicapsularis* (Sm.) Mitt. *	0.153	0.227	Poss. Thr.	**Phascum** **cuspidatum** Schreb. ex Hedw.	LC *
**Wijkia jungneri** (Broth.) H.A. Crum	0.041	0.023	Non-Thr.		

## Data Availability

Data are contained within the article.

## Supplementary Materials

The supporting information can be downloaded at: https://www.mdpi.com/article/10.3390/plants13152019/s1.

## Abbreviations

Minimum adequate model (MAM).
