# Supplementary material for: Predicting the Threat Status of Mosses Using Functional Traits
Source: Plants (Basel). 2024 Jul 23;13(15):2019. doi: 10.3390/plants13152019 (PMC11314510; doi:10.3390/plants13152019)
Supplement: Supplementary file 1 [file plants-13-02019-s001.zip › plants-2995805_Figure S1.pdf]

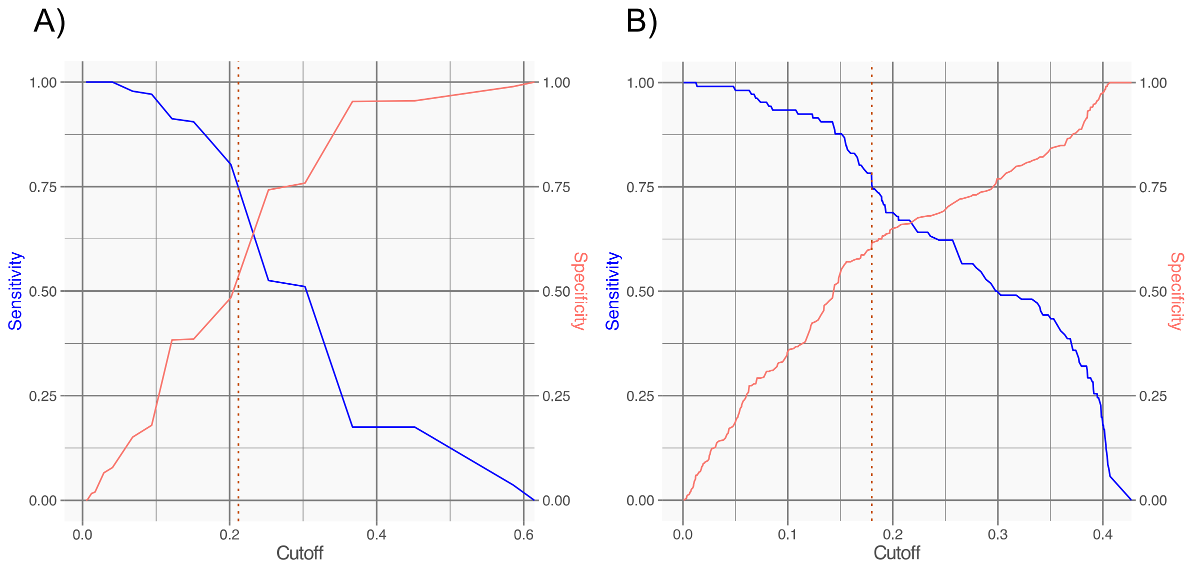

Supplementary Material Figure S1: Two-graph receiver operator characteristic curves for MAM1 (1A) and MAM2 (1B), with sensitivities and specificities plotted against the extinction risks (cutoff). The decided cutoffs were marked with a dotted line.
