# Supplementary material for: Predicting the Threat Status of Mosses Using Functional Traits
Source: Plants (Basel). 2024 Jul 23;13(15):2019. doi: 10.3390/plants13152019 (PMC11314510; doi:10.3390/plants13152019)
Supplement: Supplementary file 1 [file plants-13-02019-s001.zip › SuppMat_Table1.pdf]

Supplementary Material Table S1: Species list used for the analyses with their IUCN Red List status (RL Status): LC, NT, VU, EN, CR or EX, from the IUCN Red List (www.iucnredlist.org) or the Plants Under Pressure programme at the Natural History Museum.

Species Name	RL Status
**Acaulon dertosense** Casas, Sérgio, Cros & Brugués	EN
**Acritodon nephophilus** H. Rob.	CR
**Aloina brevirostris** (Hook. & Grev.) Kindb.	LC
**Aloina obliquifolia** (Müll.Hal.) Broth.	LC
**Aloina rigida** (Hedw.) Limpr.	LC
**Amblyodon dealbatus** (Hedw.) P. Beauv.	LC
**Amblystegium serpens** (Hedw.) Schimp.	LC
**Ambuchanania leucobryoides** (T. Yamag., Seppelt & Z. Iwats.) Seppelt & H.A. Crum ex A.J. Shaw	VU
**Amphidium lapponicum** (Hedw.) Schimp.	LC
**Anacolia laevisphaera** (Taylor) Flowers	LC
**Andoa berthelotiana** (Mont.) Ochyra	VU
**Andreaea barbarae** Luceño, Cerrejón, J. Muñoz & Maguilla	EN
**Andreaea frigida** Huebener	VU
**Andreaea megistospora** B.M.Murray	LC
**Andreaea mutabilis** Hook.f. & Wilson	LC
**Andreaea rothii** F.Weber & D.Mohr	LC
**Andreaea rupestris** Hedw.	LC
**Anoectangium aestivum** (Hedw.) Mitt.	LC
**Anomobryum concinnatum** (Spruce) Lindb.	LC
**Anomobryum julaceum** (Schrad. ex G.Gaertn., B.Mey. & Scherb.) Schimp.	LC
**Anomobryum lusitanicum** (I.Hagen ex Luisier) Thér.	VU
**Anomodon attenuatus** (Hedw.) Huebener	LC
**Anomodon longifolius** (Schleich. ex Brid.) Hartm.	LC
**Anomodon minor** (Hedw.) Lindb.	LC
**Anomodon tristis** (Ces.) Sull. & Lesq.	VU
**Anomodon viticulosus** (Hedw.) Hook. & Taylor	LC
**Antitrichia californica** Sull. ex Lesq.	LC
**Antitrichia curtipendula** (Hedw.) Brid.	LC
**Aongstroemia longipes** (Sommerf.) Bruch & Schimp.	LC
**Aplodon wormskioldii** (Hornem.) R.Br.	LC
**Archidium alternifolium** (Dicks. ex Hedw.) Schimp.	LC
**Archidium donnellii** Austin	LC
**Arctoa anderssonii** Wich.	VU
**Arctoa fulvella** (Dicks.) Bruch & Schimp.	LC
**Arctoa hyperborea** (Gunnerus ex With.) Bruch & Schimp.	VU
**Arvernella microclada** Hugonnot & Hedenäs	EN
**Aschisma cuynetii** (Bizot & R.B.Pierrot) J.Guerra & M.J.Cano	EN
**Aschisma kansanum** A.L. Andrews	LC
**Atractylocarpus alpinus** (Schimp. ex Milde) Lindb.	CR
**Atrichum crispulum** Schimp. ex Besch.	LC
**Atrichum flavisetum** Mitt.	LC
**Atrichum tenellum** (Röhl.) Bruch & Schimp.	LC
**Atrichum undulatum** (Hedw.) P.Beauv.	LC
**Aulacomnium androgynum** (Hedw.) Schwägr.	LC

**Aulacomnium palustre** (Hedw.) Schwägr.	LC
**Aulacomnium turgidum** (Wahlenb.) Schwägr.	LC
**Barbula unguiculata** Hedw.	LC
**Bartramia halleriana** Hedw.	LC
**Bartramia ithyphylla** Brid.	LC
**Bartramia pomiformis** Hedw.	LC
**Bartramia subulata** Bruch & Schimp.	EN
**Bellibarbula kurziana** P.C. Chen	NT
**Blindia acuta** (Hedw.) Bruch & Schimp.	LC
**Brachydontium trichodes** (F.Weber) Fürnr.	LC
**Brachymenium murale** Schimp. ex Besch.	VU
**Brachytheciastrum collinum** (Schleich. ex Müll.Hal.) Ignatov & Huttunen	LC
**Brachytheciastrum trachypodium** (Funck ex Brid.) Ignatov & Huttunen	LC
**Brachytheciastrum vanekii** (Šmarda) Ochyra & Żarnowiec	EN
**Brachytheciastrum velutinum** (Hedw.) Ignatov & Huttunen	LC
**Brachythecium albicans** (Hedw.) Schimp.	LC
**Brachythecium bolanderi** (Lesq.) A. Jaeger	LC
**Brachythecium campestre** (Müll.Hal.) Schimp.	LC
**Brachythecium cirrosum** (Schwägr.) Schimp.	LC
**Brachythecium erythrorrhizon** Schimp.	LC
**Brachythecium funkii** Schimp.	VU
**Brachythecium geheebii** Milde	VU
**Brachythecium laetum** (Brid.) Schimp.	LC

**Brachythecium rivulare** Schimp.	LC
**Brachythecium rutabulum** (Hedw.) Schimp.	LC
**Brachythecium salebrosum** (Hoffm. ex F.Weber & D.Mohr) Schimp.	LC
**Brachythecium turgidum** (Hartm.) Kindb.	LC
**Brachythecium udum** I.Hagen	LC
**Breutelia azorica** (Mitt.) Cardot	EN
**Bruchia vogesiaca** Schwägr.	EN
**Brymela tutezona** Crosby & B.H. Allen	CR
**Bryoerythrophyllum caledonicum** D.G.Long	VU
**Bryoerythrophyllum duellii** Blockeel	VU
**Bryoerythrophyllum ferruginascens** (Stirt.) Giacom.	LC
**Bryoerythrophyllum recurvirostrum** (Hedw.) P.C.Chen	LC
**Bryoerythrophyllum rubrum** (Jur. ex Geh.) P.C.Chen	NT
**Bryoxiphium madeirense** A.Löve & D.Löve	EN
**Bryoxiphium norvegicum** (Brid.) Mitt.	LC
**Bryum argenteum** Hedw.	LC
**Bryum austriacum** Köckinger, Holyoak & Suanjak	VU
**Bryum versicolor** A.Braun ex Bruch & Schimp.	EN
**Bucklandiella pacifica** (Ireland & J.R. Spence) Bedn.-Ochyra & Ochyra	LC
**Callicladium haldanianum** (Grev.) H.A. Crum	LC
**Callicostella pallida** (Hornsch.) Spruce	LC
**Calliergon giganteum** (Schimp.) Kindb.	LC

**Calliergon megalophyllum** Mikut.	LC
**Calliergon richardsonii** (Mitt.) Kindb.	LC
**Calliergonella cuspidata** (Hedw.) Loeske	LC
**Calymperes erosum** Müll.Hal.	LC
**Calymperes pallidum** Mitt.	LC
**Calymperes tahitense** Lindb. ex Besch.	LC
**Campyliadelphus chrysophyllus** (Brid.) R.S.Chopra	LC
**Campylium laxifolium** Engelmark & Hedenäs	LC
**Campylium longicuspis** (Lindb. & Arnell) Hedenäs	VU
**Campylium protensum** (Brid.) Kindb.	LC
**Campylium stellatum** (Hedw.) C.E.O.Jensen	LC
**Campylophyllum halleri** (Hedw.) M. Fleisch.	LC
**Campylopus atrovirens** De Not.	LC
**Campylopus comosus** (Schwägr.) Bosch & Sande Lac.	LC
**Campylopus flexuosus** (Hedw.) Brid.	LC
**Campylopus gracilis** (Mitt.) A.Jaeger	LC
**Campylopus pilifer** Brid.	LC
**Campylopus pyriformis** (Schultz) Brid.	LC
**Campylopus schimperi** Milde	LC
**Campylopus schmidii** (Müll. Hal.) A. Jaeger	LC
**Campylopus taiwanensis** akurai	LC
**Campylopus umbellatus** (Arn.) Paris	LC
**Catoscopium nigritum** (Hedw.) Brid.	LC
**Ceratodon purpureus** (Hedw.) Brid.	LC
**Chaetomitrium spinosum** E.B. Bartram	NT
**Chryso-hypnum diminutivum**	LC
**Cinclidium arcticum** (Bruch & Schimp.) Schimp.	LC
**Cinclidium stygium** Sw.	LC
**Cinclidium subrotundum** Lindb.	LC
**Cirriphyllum piliferum** (Hedw.) Grout	LC
**Claopodium whippleanum** (Sull.) Renauld & Cardot	LC
**Cleistocarpidium palustre** (Bruch & Schimp.) Ochyra & Bednarek-Ochyra	VU
**Climacium dendroides** (Hedw.) F.Weber & D.Mohr	LC
**Conostomum tetragonum** (Hedw.) Lindb.	LC
**Coscinodon cribrosus** (Hedw.) Spruce	LC
**Coscinodon horridus** (J.Muñoz & Hespanhol) Hugonnot, R.D.Porley & Ignatov	VU
**Coscinodon monchiquensis** R.D.Porley, Ochyra & Ignatova	CR
**Cratoneuron filicinum** (Hedw.) Spruce	LC
**Crossidium aberrans** Holz. & E.B. Bartram	LC
**Crossidium squamiferum** (Viv.) Jur.	LC
**Cryphaea nervosa** (Hook. & Wilson) Müll. Hal.	LC
**Cynodontium alpestre** (Wahlenb.) Milde	LC
**Cynodontium fallax** Limpr.	NT
**Cynodontium gracilescens** (F.Weber & D.Mohr) Schimp.	LC
**Cynodontium jenneri** (Schimp.) Stirt.	LC
**Cynodontium polycarpon** (Hedw.) Schimp.	LC
**Cynodontium strumiferum** (Hedw.) Lindb.	LC

**Cynodontium tenellum** (Schimp.) Limpr.	LC
**Cyrtomnium hymenophylloides** (Huebener) T.J.Kop.	LC
**Cyrtomnium hymenophyllum** (Bruch & Schimp.) Holmen	LC
**Daltonia splachnoides** (Sm.) Hook. & Taylor	LC
**Dichelyma falcatum** (Hedw.) Myrin	LC
**Dichodontium pellucidum** (Hedw.) Schimp.	LC
**Dicranella cerviculata** (Hedw.) Schimp.	LC
**Dicranella crispa** (Hedw.) Schimp.	LC
**Dicranella heteromalla** (Hedw.) Schimp.	LC
**Dicranella rotundata** (Brotherus) Takaki	NT
**Dicranella rufescens** (Dicks.) Schimp.	LC
**Dicranella schreberiana** (Hedw.) Hilf. ex H.A.Crum & L.E.Anderson	LC
**Dicranella subulata** (Hedw.) Schimp.	LC
**Dicranella varia** (Hedw.) Schimp.	LC
**Dicranodontium asperulum** (Mitt.) Broth.	LC
**Dicranodontium denudatum** (Brid.) E.Britton	LC
**Dicranodontium didymodon** (Griffith) Paris	LC
**Dicranodontium porodictyon** Cardot & Thériot	LC
**Dicranodontium uncinatum** (Harv.) A.Jaeger	LC
**Dicranoweisia cirrata** (Hedw.) Lindb. ex Milde	LC
**Dicranum acutifolium** (Lindb. & Arnell) C.E.O.Jensen	LC
**Dicranum bonjeanii** De Not.	LC
**Dicranum brevifolium** (Lindb.) Lindb.	LC
**Dicranum crispifolium** Müll.Hal.	LC
**Dicranum drummondii** Müll.Hal.	LC
**Dicranum elongatum** Schleich. ex Schwägr.	LC
**Dicranum flagellare** Hedw.	LC
**Dicranum fragilifolium** Lindb.	LC
**Dicranum fulvum** Hook.	LC
**Dicranum fuscescens** Turner	LC
**Dicranum groenlandicum** Brid.	LC
**Dicranum leiodontium** Cardot	LC
**Dicranum leioneuron** Kindb.	LC
**Dicranum lorifolium** Mitt.	LC
**Dicranum majus** Turner	LC
**Dicranum montanum** Hedw.	LC
**Dicranum ontariense** Peterson	LC
**Dicranum polysetum** Sw. ex anon.	LC
**Dicranum scoparium** Hedw.	LC
**Dicranum spadiceum** J.E.Zetterst.	LC
**Dicranum spurium** Hedw.	LC
**Dicranum tauricum** Sapjegin	LC
**Dicranum undulatum** Schrad. ex Brid.	LC
**Dicranum viride** (Sull. & Lesq.) Lindb.	LC
**Didymodon acutus** (Brid.) K.Saito	LC
**Didymodon australasiae** (Hook. & Grev.) R.H. Zander	LC
**Didymodon bistratosus** Hébr. & R.B. Pierrot	LC
**Didymodon eckeliae** R.H. Zander	LC
**Didymodon fallax** (Hedw.) R.H. Zander	LC

**Didymodon ferrugineus** (Schimp. ex Besch.) M.O. Hill	LC
**Didymodon giganteus** (Funck) Jur.	LC
**Didymodon maximus** (Syed & Crundw.) M.O. Hill	VU
**Didymodon nicholsonii** Culm.	LC
**Didymodon norrisii** Zander	LC
**Didymodon rigidulus** Hedw.	LC
**Didymodon subandreaeoides** (Kindb.) R.H. Zander	LC
**Didymodon tophaceus** (Brid.) Lisa	LC
**Didymodon umbrosus** (Müll. Hal.) R.H. Zander	LC
**Diphyscium foliosum** (Hedw.) D.Mohr	LC
**Discelium nudum** (Dicks.) Brid.	LC
**Distichium capillaceum** (Hedw.) Bruch & Schimp.	LC
**Distichium inclinatum** (Hedw.) Bruch & Schimp.	LC
**Distichophyllum carinatum** Dixon & W.E.Nicholson	EN
**Ditrichum cornubicum** Paton	CR
**Ditrichum flexicaule** (Schwägr.) Brockm.	LC
**Ditrichum heteromallum** (Hedw.) E.Britton	LC
**Ditrichum lineare** (Sw.) Lindb.	LC
**Ditrichum plumbicola** Crundw.	EN
**Ditrichum pusillum** (Hedw.) Hampe	LC
**Donrichardsia macroneuron** (Grout) H.A. Crum & L.E. Anderson	VU
**Drepanocladus aduncus** (Hedw.) Warnst.	LC
**Drepanocladus arcticus** (R.S.Williams) Hedenäs	NT
**Drepanocladus polygamus** (Schimp.) Hedenäs	LC

**Echinodium renauldii** (Cardot) Broth.	EN
**Echinodium setigerum** (Mitt.) Jur.	EN
**Echinodium spinosum** (Mitt.) Jur.	EN
**Encalypta affinis** R.Hedw.	LC
**Encalypta alpina** Sm.	LC
**Encalypta brevipes** Schljakov	EN
**Encalypta ciliata** Hedw.	LC
**Encalypta procera** Bruch	LC
**Encalypta rhaptocarpa** Schwägr.	LC
**Encalypta vulgaris** Hedw.	LC
**Entodon beyrichii** (Schwaegrichen) Müll.Hal.	LC
**Entodon concinnus** (De Not.) Paris	LC
**Entodon geminidens** (Bescherelle) Paris	NT
**Entodon schleicheri** (Schimp.) Demet.	LC
**Entosthodon attenuatus** (Dicks.) Bryhn	LC
**Entosthodon fascicularis** (Hedw.) Müll.Hal.	LC
**Ephemerum crassinervium** (Schwägr.) Hampe	NT
**Ephemerum serratum** (Schreb. ex Hedw.) Hampe	LC
**Eucladium verticillatum** (With.) Bruch & Schimp.	LC
**Eurhynchiastrum pulchellum** (Hedw.) Ignatov & Huttunen	LC
**Exsertotheca baetica** (J. Guerra, J.F. Jiménez & J.A. Jiménez) Draper, González-Mancebo, O. Werner, J. Patiño & Ros	EN
**Exsertotheca intermedia** (Brid.) S. Olsson, Enroth & D. Quandt	VU
**Fissidens adianthoides** Hedw.	LC
**Fissidens arcticus** Bryhn	EN
**Fissidens asplenioides** Hedw.	LC

**Fissidens azoricus** (P.de la Varde) Bizot	CR
**Fissidens bryoides** Hedw.	LC
**Fissidens crispus** Mont.	LC
**Fissidens dubius** P.Beauv.	LC
**Fissidens exilis** Hedw.	LC
**Fissidens fontanus** (Bach.Pyl.) Steud.	LC
**Fissidens grandifrons** Brid.	LC
**Fissidens hydropogon** Spruce ex Mitt.	CR
**Fissidens involutus** Wilson ex Mitt.	LC
**Fissidens jansenii** Sérgio & Pursell	CR
**Fissidens microstictus** Dixon & Luisier	EX
**Fissidens nobreganus** Dixon & Luisier	EN
**Fissidens osmundoides** Hedw.	LC
**Fissidens taxifolius** Hedw.	LC
**Fissidens tosaensis**	LC
**Flabellidium spinosum** Herzog	EX
**Fontinalis antipyretica** Hedw.	LC
**Fontinalis hypnoides** C.Hartm.	LC
**Fontinalis sphagnifolia** (Müll.Hal.) Wijk & Margadant	LC
**Funaria hygrometrica** Hedw.	LC
**Funaria microstoma** Bruch ex Schimp.	LC
**Funaria polaris** Bryhn	LC
**Imbribryum tenuisetum** (Limpr.) D. Bell & Holyoak	LC
**Glyphomitrium formosanum** Iwatsuki	NT
**Gradsteinia torrenticola** Ochyra, C. Schmidt & Bültmann	VU
**Grimmia alpestris** (F.Weber & D.Mohr) Schleich.	LC
**Grimmia anodon** Bruch & Schimp.	LC

**Grimmia anomala** Hampe ex Schimp.	LC
**Grimmia atrata** Miel. ex Hornsch.	LC
**Grimmia caespiticia** (Brid.) Jur.	LC
**Grimmia crinitoleucophaea** Cardot	LC
**Grimmia curviseta** Bouman	VU
**Grimmia donniana** Sm.	LC
**Grimmia elatior** Bruch ex Bals.-Criv. & De Not.	LC
**Grimmia elongata** Kaulf.	LC
**Grimmia funalis** (Schwägr.) Bruch & Schimp.	LC
**Grimmia hartmanii** Schimp.	LC
**Grimmia incurva** Schwägr.	LC
**Grimmia laevigata** (Brid.) Brid.	LC
**Grimmia lisae** De Not.	LC
**Grimmia longirostris** Hook.	LC
**Grimmia montana** Bruch & Schimp.	LC
**Grimmia muehlenbeckii** Schimp.	LC
**Grimmia nevadensis** Greven	LC
**Grimmia orbicularis** Bruch ex Wilson	LC
**Grimmia pulvinata** (Hedw.) Sm.	LC
**Grimmia ramondii** (Lam. & DC.) Margad.	LC
**Grimmia reflexidens** Müll.Hal.	LC
**Grimmia torquata** Drumm.	LC
**Grimmia trichophylla** Grev.	LC
**Grimmia unicolor** Hook.	LC
**Gymnobarbula bicolor** (Bruch & Schimp.) Jan Kučera	VU
**Gymnostomum aeruginosum** Sm.	LC
**Gymnostomum calcareum** Nees & Hornsch.	LC
**Gymnostomum viridulum** Brid.	LC
**Gyroweisia tenuis** (Hedw.) Schimp.	LC

**Hedenasiastrum percurrens** (Hedenäs) Ignatov & Vanderp.	EN
**Hedwigia ciliata** (Hedw.) P.Beauv.	LC
**Hedwigia stellata** Hedenäs	LC
**Hennediella heimii** (Hedw.) R.H.Zander	LC
**Herzogiella adscendens** (Lindberg) Iwatsuki & W. B. Schofield	LC
**Herzogiella seligeri** (Brid.) Z. Iwats.	LC
**Herzogiella striatella** (Brid.) Z. Iwats.	LC
**Heterocladium dimorphum** (Brid.) Schimp.	LC
**Homalia trichomanoides** (Hedw.) Schimp.	LC
**Homalia webbiana** (Mont.) Schimp.	EN
**Homalothecium aureum** (Spruce) H. Rob.	LC
**Homalothecium sericeum** (Hedw.) Schimp.	LC
**Homomallium incurvatum** (Schrad. ex Brid.) Loeske	LC
**Hygroamblystegium varium** (Hedw.) Mönk.	LC
**Hygrohypnum alpinum** (Lindb.) Loeske	LC
**Hygrohypnum bestii** (Renauld & Bryhn) Brotherus	LC
**Pseudohygrohypnum eugyrium** (Schimp.) Kanda	LC
**Hygrohypnum luridum** (Hedw.) Jenn.	LC
**Hygrohypnum polare** (Lindb.) Loeske	LC
**Hylocomiastrum umbratum** (Hedw.) M.Fleisch. ex Broth.	LC
**Hylocomium splendens** (Hedw.) Schimp.	LC
**Hymenostylium gracillimum** (Nees & Hornsch.) Köckinger & J.Kučera	EN

**Hymenostylium recurvirostrum** (Hedw.) Dixon	LC
**Hyophila involuta** (Hook.) A. Jaeger	LC
**Hyophila spathulata** (Harvey) Jaeger	LC
**Hypnella punctata** Brotherus	CR
**Hypnodontopsis apiculata** Z. Iwats. & Nog.	VU
**Hypnum andoi** A.J.E. Sm.	LC
**Hypnum bambergeri** Schimp.	LC
**Hypnum callichroum** Brid.	LC
**Hypnum cupressiforme** Hedw.	LC
**Hypnum curvifolium**	LC
**Hypnum fertile** Sendtn.	CR
**Hypnum hamulosum** Schimp.	LC
**Hypnum jutlandicum** Holmen & E. Warncke	LC
**Hypnum pallescens** (Hedw.) P. Beauv.	LC
**Hypnum recurvatum** (Lindb. & Arnell) Kindb.	LC
**Imbribryum alpinum** (Huds. ex With.) N.Pedersen	LC
**Imbribryum mildeanum** (Jur.) J.R.Spence	LC
**Imbribryum muehlenbeckii** (Bruch & Schimp.) N.Pedersen	LC
**Isopterygiopsis muelleriana** (Schimp.) Z. Iwats.	LC
**Isopterygiopsis pulchella** (Hedw.) Z. Iwats.	LC
**Isothecium alopecuroides** (Lam. ex Dubois) Isov.	LC
**Isothecium montanum** Draper, Hedenäs, M.Stech, T.Lopes & Sim-Sim	CR
**Isothecium myosuroides** Brid.	LC
**Isothecium prolixum** (Mitt.) M.Stech, Sim-Sim, Tangney & D.Quandt	VU

**Isothecium stoloniferum** Bridel	LC
**Iwatsukiella leucotricha** (Mitt.) W.R. Buck & H.A. Crum	LC
**Jaffueliobryum arsenei** (Thér.) Thér.	EN
**Kiaeria blyttii** (Bruch & Schimp.) Broth.	LC
**Kiaeria falcata** (Hedw.) I.Hagen	LC
**Kiaeria glacialis** (Berggr.) I.Hagen	LC
**Kiaeria starkei** (F.Weber & D.Mohr) I.Hagen	LC
**Kindbergia praelonga** (Hedw.) Ochyra	LC
**Lepidopilum grevilleanum** Spruce	CR
**Lepidopilum wallisii** Müll. Hal.	CR
**Leptobryum pyriforme** (Hedw.) Wilson	LC
**Leptodictyum riparium** (Hedw.) Warnst.	LC
**Leptodon corsicus** Enroth, A. Sotiaux, D. Quandt & Vanderp.	CR
**Leptodon longisetus** Mont.	VU
**Lescuraea saxicola** (Schimp.) Molendo	LC
**Leskea polycarpa** Hedw.	LC
**Leucobryum albidum** (Brid. ex P.Beauv.) Lindb.	LC
**Leucobryum glaucum** (Hedw.) Ångstr.	LC
**Leucobryum humillimum** Cardot	LC
**Leucobryum juniperoideum** (Brid.) Müll.Hal.	LC
**Leucodon treleasei** (Cardot) Paris	VU
**Leucoperichaetium eremophilum** Magill	VU
**Lewinskya lamyana** F.Lara, Garilleti, Draper & Mazimpaka	CR
**Limbella fryei** (R.S. Williams) Ochyra	CR
**Loeskeobryum brevirostre** (Brid.) M. Fleisch.	LC
**Lorentziella imbricata** (Mitten) Brotherus	LC
**Mamillariella geniculata** Laz.	EN
**Meesia uliginosa** Hedw.	LC
**Merrilliobryum fabronioides** Broth.	EN
**Microbryum floerkeanum** (F.Weber & D.Mohr) Schimp.	LC
**Microbryum longipes** (J. Guerra, J.J. Martínez & Ros) R.H. Zander	VU
**Microcampylopus laevigatus** (Thér.) Giese & J.-P.Frahm	NT
**Mielichhoferia mielichhoferiana** (Funck) Loeske	LC
**Mitrobryum koelzii** H.Rob.	EN
**Mnium blyttii** Bruch & Schimp.	LC
**Mnium hornum** Hedw.	LC
**Mnium lycopodioides** Schwägr.	LC
**Mnium marginatum** (Dicks. ex With.) P.Beauv.	LC
**Mnium spinosum** (Voit) Schwägr.	LC
**Mnium spinulosum** Bruch & Schimp.	LC
**Mnium stellare** Hedw.	LC
**Mnium thomsonii** Schimp.	LC
**Molendoa hornschuchiana** (Hook.) Lindb. ex Limpr.	LC
**Molendoa schliephackei** (Limpr.) R.H. Zander	EN
**Molendoa taeniatifolia** Herzog	EN
**Neckera menziesii** Drumm.	LC
**Neckera pennata** Hedw.	LC
**Neckeropsis pocsii** Enroth & Magill	CR
**Neckeropsis undulata**	LC
**Neomacounia nitida**	EX

**Nobregaea latinervis** Hedenäs	EX
**Nogopterium gracile** (Hedw.) Crosby & W.R. Buck	LC
**Ochyraea tatrensis** Vána	CR
**Oligotrichum hercynicum** (Hedw.) Lam. & DC.	LC
**Oncophorus virens** (Hedw.) Brid.	LC
**Oncophorus wahlenbergii** Brid.	LC
**Orthodontopsis bardunovii** Ignatov & B.C. Tan	EN
**Orthothecium intricatum** (Hartm.) Schimp.	LC
**Orthotrichum alpestre** Hornsch. ex Bruch & Schimp.	LC
**Orthotrichum anomalum** Hedw.	LC
**Orthotrichum casasianum** F. Lara, Garilleti & Mazimpaka	CR
**Orthotrichum cupulatum** Brid.	LC
**Orthotrichum dentatum** T.Kiebacher & Lüth	VU
**Orthotrichum diaphanum** Brid.	LC
**Orthotrichum exiguum** Sull.	LC
**Orthotrichum flowersii** Vitt	LC
**Orthotrichum handiense** F. Lara, Garilleti & Mazimpaka	CR
**Orthotrichum pallens** Bruch ex Brid.	LC
**Orthotrichum pulchellum** Brunt.	LC
**Orthotrichum pumilum** Sw.	LC
**Orthotrichum rivulare** Turner	LC
**Orthotrichum shawii** Wilson	LC
**Orthotrichum stramineum** Hornsch. ex Brid.	LC
**Orthotrichum tenellum** Bruch ex Brid.	LC
**Orthotrichum truncato-dentatum** Müll. Hal.	EN
**Ozobryum ogalalense** G.L. Merr.	CR
**Paludella squarrosa** (Hedw.) Brid.	LC
**Palustriella falcata** (Brid.) Hedenäs	LC
**Paraleucobryum enerve** (Thed.) Loeske	LC
**Paraleucobryum longifolium** (Ehrh. ex Hedw.) Loeske	LC
**Paraleucobryum sauteri** (Bruch & Schimp.) Loeske	NT
**Pelekium atlanticum** (Hedenäs) Hedenäs	EN
**Philonotis capillaris** Lindb.	LC
**Philonotis fontana** (Hedw.) Brid.	LC
**Philonotis seriata** Mitt.	LC
**Physcomitrella patens** (Hedw.) Bruch & Schimp.	LC
**Physcomitrium arenicola** Laz.	EN
**Physcomitrium collenchymatum** Gier	LC
**Physcomitrium hookeri** Hampe	LC
**Physcomitrium pyriforme** (Hedw.) Hampe	LC
**Pinnatella limbata** Dixon	CR
**Plagiomnium cuspidatum** (Hedw.) T.J.Kop.	LC
**Plagiomnium ellipticum** (Brid.) T.J.Kop.	LC
**Plagiomnium medium** (Bruch & Schimp.) T.J.Kop.	LC
**Plagiomnium rostratum** (Schrad.) T.J.Kop.	LC
**Plagiomnium undulatum** (Hedw.) T.J.Kop.	LC
**Plagiopus oederianus** (Sw.) H.A.Crum & L.E.Anderson	LC
**Plagiothecium berggrenianum** Frisvoll	VU
**Plagiothecium cavifolium** (Brid.) Z. Iwats.	LC

**Plagiothecium denticulatum** (Hedw.) Schimp.	LC
**Plagiothecium laetum** Schimp.	LC
**Plagiothecium latebricola** Schimp.	LC
**Plagiothecium piliferum** (Sw.) Schimp.	LC
**Platydictya jungermannioides** (Brid.) H.A.Crum	LC
**Platydictya subtilis** (Hedw.) H.A. Crum	LC
**Platygyrium repens** (Brid.) Schimp.	LC
**Pleuridium acuminatum** Lindb.	LC
**Pleuridium lindigianum** (Hampe) S.P. Churchill	EN
**Pleuridium subulatum** (Hedw.) Rabenh.	LC
**Pogonatum dentatum** (Menzies ex Brid.) Brid.	LC
**Pogonatum urnigerum** (Hedw.) P. Beauv.	LC
**Pohlia andalusica** (Höhn.) Broth.	LC
**Pohlia annotina** (Hedw.) Lindb.	LC
**Pohlia beringiensis** A.J.Shaw	CR
**Pohlia bulbifera** (Warnst.) Warnst.	LC
**Pohlia camptotrachela** (Renauld & Cardot) Broth.	LC
**Pohlia cruda** (Hedw.) Lindb.	LC
**Pohlia crudoides** (Sull. & Lesq.) Broth.	LC
**Pohlia drummondii** (Müll.Hal.) A.L.Andrews	LC
**Pohlia elongata** Hedw.	LC
**Pohlia erecta** Lindb.	EN
**Pohlia filum** (Schimp.) Mårtensson	LC
**Pohlia flexuosa** Hook.	LC
**Pohlia lescuriana** (Sull.) Ochi	LC
**Pohlia longibracteata** Brotherus in Röll	LC
**Pohlia longicolla** (Hedw.) Lindb.	LC
**Pohlia ludwigii** (Spreng. ex Schwägr.) Broth.	LC
**Pohlia melanodon** (Brid.) A.J.Shaw	LC
**Pohlia nutans** (Hedw.) Lindb.	LC
**Pohlia obtusifolia** (Vill. ex Brid.) L.F.Koch	LC
**Pohlia proligera** (Kindb.) Lindb. ex Broth.	LC
**Pohlia wahlenbergii** (F.Weber & D.Mohr) A.L.Andrews	LC
**Polytrichastrum alpinum** (Hedw.) G.L. Sm.	LC
**Polytrichum commune** Hedw.	LC
**Polytrichum hyperboreum** R. Br.	LC
**Polytrichum jensenii** I.Hagen	LC
**Polytrichum juniperinum** Hedw.	LC
**Polytrichum piliferum** Hedw.	LC
**Polytrichum strictum** Menzies ex Brid.	LC
**Polytrichum swartzii** Hartm.	LC
**Pseudephemerum nitidum** (Hedw.) Loeske	LC
**Pseudocampylium radicale** (P.Beauv.) Vanderp. & Hedenäs	LC
**Pseudochorisodontium gymnostomum** (Mitt.) C. Gao, Vitt, X. Fu & T. Cao	LC
**Pseudochorisodontium mamillosum** (Gao Chien & Aur Zhi-wen) Gao Chien	VU
**Pseudoleskeella rupestris** (Berggr.) Hedenäs & L. Söderstr.	LC
**Pseudoleskeella tectorum** (Funck ex Brid.) Kindb. ex Broth.	LC
**Pseudoscleropodium purum** (Hedw.) M.Fleisch.	LC

**Pseudotaxiphyllum elegans** (Brid.) Z. Iwats.	NT
**Psilopilum cavifolium** (Wilson) I. Hagen	LC
**Psilopilum laevigatum** (Wahlenb.) Lindb.	LC
**Pterigynandrum filiforme** Hedw.	LC
**Pterygoneurum kozlovii** Laz.	LC
**Pterygoneurum lamellatum** (Lindb.) Jur.	LC
**Pterygoneurum subsessile** (Brid.) Jur.	LC
**Ptychomitrium incurvum** (Schwägr.) Spruce	LC
**Ptychomitrium serratum** Bruch & Schimp.	LC
**Ptychostomum arcticum** (R.Br.) J.R.Spence ex Holyoak & N.Pedersen	LC
**Ptychostomum cernuum** (Hedw.) Hornsch.	LC
**Ptychostomum creberrimum** (Taylor) J.R.Spence & H.P.Ramsay	LC
**Ptychostomum longisetum** (Blandow ex Schwägr.) J.R.Spence	LC
**Ptychostomum meesioides**	LC
**Ptychostomum pallens** (Sw.) J.R.Spence	LC
**Ptychostomum pallescens** (Schleich. ex Schwägr.) J.R.Spence	LC
**Ptychostomum pseudotriquetrum** (Hedw.) J.R.Spence & H.P.Ramsay ex Holyoak & N.Pedersen	LC
**Pylaisia polyantha** (Hedw.) Schimp.	LC
**Pylaisia selwynii** Kindb.	LC
**Pyramidula tetragona** (Brid.) Brid.	LC
**Pyrrhobryum spiniforme** (Hedw.) Mitt.	LC
**Racomitrium lanuginosum** (Hedw.) Brid.	LC

**Racomitrium lusitanicum** Ochyra & Sérgio	EN
**Racomitrium nivale** (Köckinger, Bedn.-Ochyra & Ochyra) Köckinger	VU
**Renauldia lycopodioides** Bizot	EN
**Rhabdoweisia crenulata** (Mitt.) H.Jameson	LC
**Rhabdoweisia crispata** (Dicks. ex With.) Lindb.	LC
**Rhachithecium perpusillum** (Thwaites & Mitt.) Broth.	LC
**Rhizomnium appalachianum** T.J. Kop.	LC
**Rhizomnium gracile** T.J.Kop.	LC
**Rhizomnium magnifolium** (Horik.) T.J.Kop.	LC
**Rhizomnium pseudopunctatum** (Bruch & Schimp.) T.J.Kop.	LC
**Rhizomnium punctatum** (Hedw.) T.J.Kop.	LC
**Rhodobryum ontariense** (Kindb.) Kindb.	LC
**Rhodobryum roseum** (Hedw.) Limpr.	NT
**Rhynchostegiella bourgaeana** (Mitt.) Broth.	EN
**Rhynchostegiella trichophylla** Dirkse & Bouman	VU
**Rhynchostegium confusum** Cezón, J.Muñoz, Hedenäs & Huttunen	VU
**Rhynchostegium serrulatum** (Hedw.) Austin	LC
**Rhynchostegium strongylense** (Bott.) W.R.Buck & Privitera	EN
**Rhytidiadelphus loreus** (Hedw.) Warnst.	LC
**Rhytidiadelphus squarrosus** (Hedw.) Warnst.	LC
**Rhytidiadelphus subpinnatus** (Lindb.) T.J. Kop.	LC

**Rhytidiadelphus triquetrus** (Hedw.) Warnst.	LC
**Rhytidium rugosum** (Hedw.) Kindb.	LC
**Sanionia nivalis** Hedenäs	LC
**Sanionia orthothecioides** (Lindb.) Loeske	LC
**Sanionia uncinata** (Hedw.) Loeske	LC
**Sarmentypnum exannulatum** (Schimp.) Hedenäs	LC
**Sarmentypnum sarmentosum** (Wahlenb.) Tuom. & T.J.Kop.	LC
**Schistidium apocarpum** (Hedw.) Bruch & Schimp.	LC
**Schistidium atrofuscum** (Schimp.) Limpr.	LC
**Schistidium boreale** Poelt	LC
**Schistidium bryhnii** I.Hagen	VU
**Schistidium confertum** (Funck) Bruch & Schimp.	LC
**Schistidium crassipilum** H.H.Blom	LC
**Schistidium flaccidum** (De Not.) Ochyra	LC
**Schistidium flexipile** (Lindb. ex Broth.) G.Roth	LC
**Schistidium frisvollianum** H.H.Blom	VU
**Schistidium grande** Poelt	VU
**Schistidium grandirete** H.H.Blom	LC
**Schistidium holmenianum** Steere & Brassard	LC
**Schistidium occidentale** (E.Lawton) S.P.Churchill	LC
**Schistidium papillosum** Culm.	LC
**Schistidium pulchrum** H.H.Blom	LC
**Schistidium rivulare** (Brid.) Podp.	LC
**Schistidium robustum** (Nees & Hornsch.) H.H.Blom	LC
**Schistidium spinosum** H.H.Blom & Lüth	CR
**Schistidium strictum** (Turner) Loeske ex Mårtensson	LC
**Schistidium subjulaceum** H.H.Blom	LC
**Schistidium tenerum** (J.E.Zetterst.) Nyholm	LC
**Schistidium trichodon** (Brid.) Poelt	LC
**Schistidium venetum** H.H.Blom	EN
**Schizymenium pontevedrense** (Luisier) Sérgio, Casas, Cros & Brugués	VU
**Sciaromiopsis sinensis** (Broth.) Broth.	EN
**Sciuro-hypnum glaciale** (Schimp.) Ignatov & Huttunen	LC
**Sciuro-hypnum ornellanum** (Molendo) Ignatov & Huttunen	LC
**Sciuro-hypnum plumosum** (Hedw.) Ignatov & Huttunen	LC
**Sciuro-hypnum populeum** (Hedw.) Ignatov & Huttunen	LC
**Sciuro-hypnum reflexum** (Starke) Ignatov & Huttunen	LC
**Sciuro-hypnum starkei** (Brid.) Ignatov & Huttunen	LC
**Scleropodium cespitans** (Müll.Hal.) L.F.Koch	LC
**Scopelophila ligulata** (Spruce) Spruce	LC
**Scorpidium cossonii** (Schimp.) Hedenäs	LC
**Scorpidium scorpioides** (Hedw.) Limpr.	LC
**Seligeria acutifolia** Lindb.	LC
**Seligeria brevifolia** (Lindb.) Lindb. & Arnell	LC
**Seligeria calcarea** (Hedw.) Bruch & Schimp.	LC
**Seligeria carniolica** (Breidl. & Beck) Nyholm	EN
**Seligeria donniana** (Sm.) Müll.Hal.	VU

**Seligeria irrigata** (H.K.G.Paul) Ochyra & Gos	VU
**Seligeria oelandica** C.E.O.Jensen & Medelius	LC
**Sematophyllum adnatum** (Michx.) E. Britton	LC
**Skottsbergia paradoxa** Cardot	EN
**Sphagnum affine** Renauld & Cardot	LC
**Sphagnum angermanicum** Melin	LC
**Sphagnum angustifolium** (Russow) C.E.O. Jensen	LC
**Sphagnum annulatum** H. Lindb. ex Warnst.	LC
**Sphagnum antioquiense** H.A. Crum	CR
**Sphagnum arcticum** Flatberg & Frisvoll	LC
**Sphagnum austinii** Sull.	LC
**Sphagnum balticum** (Russow) C.E.O. Jensen	LC
**Sphagnum beothuk** R.E.Andrus	CR
**Sphagnum boyacanum** H.A. Crum	CR
**Sphagnum brasiliense** Warnst.	EN
**Sphagnum capillifolium** (Ehrh.) Hedw.	LC
**Sphagnum centrale** C.E.O. Jensen	CR
**Sphagnum cleefii** H.A. Crum	CR
**Sphagnum compactum** Lam. & DC.	LC
**Sphagnum contortum** Schultz	LC
**Sphagnum cuspidatum** Ehrh. ex Hoffm.	CR
**Sphagnum diblastoides** H.A. Crum	CR
**Sphagnum falcatulum** Besch.	LC
**Sphagnum fallax** H. Klinggr.	LC
**Sphagnum fimbriatum** Wilson	LC

**Sphagnum flavicomans** (Cardot) Warnst.	LC
**Sphagnum flexuosum** Dozy & Molk.	LC
**Sphagnum fuscum** (Schimp.) H. Klinggr.	LC
**Sphagnum girgensohnii** Russ.	LC
**Sphagnum inundatum** Russow	LC
**Sphagnum jensenii** H.Lindb.	LC
**Sphagnum lenense** H. Lindb. ex L.I. Savicz	VU
**Sphagnum lescurii** Sull.	LC
**Sphagnum lewisii** H.A. Crum	VU
**Sphagnum lindbergii** Schimp.	LC
**Sphagnum majus** (Russow) C.E.O. Jensen	LC
**Sphagnum molle** Sull.	CR
**Sphagnum nitidulum** Warnst.	CR
**Sphagnum novocaledoniae** Paris & Warnst.	VU
**Sphagnum pacificum** Flatberg	LC
**Sphagnum palustre** L.	LC
**Sphagnum papillosum** Lindb.	LC
**Sphagnum platyphyllum** (Lindb. ex Braithw.) Sull. ex Warnst.	LC
**Sphagnum portoricense** Hampe	LC
**Sphagnum pylaesii** Brid.	LC
**Sphagnum recurvum** P.Beauv.	LC
**Sphagnum riparium** Ångstr.	LC
**Sphagnum rubellum** Wilson	LC
**Sphagnum rubiginosum** Flatberg	LC
**Sphagnum russowii** Warnst.	CR
**Sphagnum santanderense** H.A. Crum	CR
**Sphagnum sonsonense** H.A. Crum	EN
**Sphagnum squarrosum** Crome in Hoppe	LC

**Sphagnum strictum** Sull.	LC
**Sphagnum subfulvum** Sjörs	LC
**Sphagnum subhomophyllum** H. Crum	CR
**Sphagnum subnitens** Russow & Warnst.	LC
**Sphagnum subsecundum** Nees in Sturm.	VU
**Sphagnum sumapazense** H.A. Crum	VU
**Sphagnum tenellum** (Brid.) Brid.	LC
**Sphagnum teres** (Schimp.) Ångstr. in Hartm.	LC
**Sphagnum troendelagicum** Flatberg	EN
**Sphagnum warnstorfii** Russow	LC
**Sphagnum wulfianum** Girg.	LC
**Splachnum ampullaceum** Hedw.	LC
**Splachnum luteum** Hedw.	EN
**Splachnum melanocaulon** (Wahlenb.) Schwägr.	EN
**Splachnum pensylvanicum** (Brid.) Grout ex H.A. Crum	LC
**Splachnum rubrum** Hedw.	LC
**Splachnum sphaericum** Hedw.	LC
**Splachnum vasculosum** Hedw.	CR
**Stegonia latifolia** (Schwägr.) Venturi ex Broth.	LC
**Straminergon stramineum** (Dicks. ex Brid.) Hedenäs	LC
**Symblepharis oncophoroides** Broth.	VU
**Syntrichia laevipila** Brid.	LC
**Syntrichia montana** Nees	LC
**Syntrichia norvegica** F. Weber	LC
**Syntrichia papillosa** (Wilson) Jur.	VU
**Syntrichia papillosissima** (Copp.) Loeske	LC
**Syrrhopodon flammeonervis** Müll. Hal.	LC
**Takakia ceratophylla** (Mitt.) Grolle	LC

**Taxitheliella richardsii** Dixon	CR
**Taxithelium planum** (Brid.) Mitt.	LC
**Tayloria hornschuchii** (Grev. & Arn.) Broth.	LC
**Tayloria lingulata** (Dicks.) Lindb.	LC
**Tayloria serrata** (Hedw.) Bruch & Schimp.	LC
**Tetraphis pellucida** Hedw.	LC
**Tetraplodon angustatus** (Hedw.) Bruch & Schimp.	EN
**Tetraplodon blyttii** Frisvoll	EN
**Tetraplodon mnioides** (Hedw.) Bruch & Schimp.	LC
**Tetraplodon urceolatus** (Hedw.) Bruch & Schimp.	VU
**Tetrastichium fontanum** (Mitt.) Cardot	VU
**Tetrodontium repandum** (Funck) Schwägr.	LC
**Thamnobryum angustifolium** (Holt) Crundw.	CR
**Thamnobryum cataractarum** N.Hodgetts & Blockeel	CR
**Thamnobryum fernandesii** Sérgio	VU
**Thamnobryum neckeroides** (Hook.) E. Lawton	EN
**Thamnobryum rudolphianum** Mastracci	EN
Thelia lescurii	LC
**Thuidium delicatulum** (Hedw Schimp.	LC
**Thuidium recognitum** (Hedw.) Lindb.	LC
**Thuidium tamariscinum** (Hedw.) Schimp.	LC
**Timmia austriaca** Hedw.	LC
**Timmia megapolitana** Hedw.	LC
**Timmia sibirica** Lindb. & Arnell	LC
**Timmiella anomala** (Bruch & Schimp.) Limpr.	LC

**Tomentypnum nitens** (Hedw.) Loeske	LC
**Tortella alpicola** Dixon	LC
**Tortella flavovirens** (Bruch) Broth.	LC
**Tortella fragilis** (Hook. & Wilson) Limpr.	LC
**Tortella humilis** (Hedw.) Jenn.	LC
**Tortella inclinata** (R. Hedw.) Limpr.	LC
**Tortella limbata** (Schiffn.) Geh. & Herzog	VU
**Tortella tortuosa** (Schrad. ex Hedw.) Limpr.	LC
**Tortula amplexa** (Lesq.) Steere	LC
**Tortula bolanderi** (Lesq.) M. Howe	LC
**Tortula californica** E.B. Bartram	LC
**Tortula cernua** (Huebener) Lindb.	LC
**Tortula guepinii** (Bruch & Schimp.) Broth.	LC
**Tortula hoppeana** (Schultz) Ochyra	LC
**Tortula inermis** (Brid.) Mont.	LC
**Tortula leucostoma** (R. Br.) Hook. & Grev.	LC
**Tortula mucronifolia** Schwägr.	LC
**Tortula muralis** Hedw.	LC
**Tortula systylia** (Schimp.) Lindb.	LC
**Trematodon laetevirens** Hakelier & J.-P.Frahm	VU
**Trematodon longicollis** Michx.	CR
**Trematodon perssoniorum** P.Allorge & Thér. ex V.Allorge	CR

**Trichodon cylindricus** (Hedw.) Schimp.	LC
**Trichosteleum mastopomatoides** S.P. Churchill & I. Sastre	CR
**Trichostomum brachydontium** Bruch	LC
**Trichostomum hattorianum** B.C. Tan & Z. Iwats.	LC
**Tuerckheimia svihlae** (E.B. Bartram) R.H. Zander	LC
**Ulota coarctata** (P. Beauv.) Hammar	LC
**Ulota crispa** (Hedw.) Brid.	LC
**Ulota curvifolia** (Wahlenb.) Sw.	LC
**Ulota drummondii** (Hook. & Grev.) Brid.	LC
**Ulota hutchinsiae** (Sm.) Hammar	LC
**Ulota macrospora** E.Bauer & Warnst.	EN
**Warnstorfia fluitans** (Hedw.) Loeske	LC
**Warnstorfia pseudostraminea** (Müll. Hal.) Tuom. & T.J. Kop.	LC
**Weisiopsis anomala** (Broth. & Paris ex Cardot) Broth.	LC
**Weissia brachycarpa** (Nees & Hornsch.) Jur.	LC
**Weissia controversa** Hedw.	LC
**Weissia squarrosa** (Nees & Hornsch.) Müll. Hal.	VU
**Zygodon conoideus** (Dicks.) Hook. & Taylor	LC
**Zygodon gracilis** Wilson	LC
**Zygodon reinwardtii** (Hornsch.) A. Braun	LC
**Zygodon viridissimus** (Dicks.) Brid.	LC
