# Supplementary material for: Predicting the Threat Status of Mosses Using Functional Traits
Source: Plants (Basel). 2024 Jul 23;13(15):2019. doi: 10.3390/plants13152019 (PMC11314510; doi:10.3390/plants13152019)
Supplement: Supplementary file 1 [file plants-13-02019-s001.zip › SuppMat_Tables2A-2C.pdf]

**Supplementary Material Table S2:** The functional traits used for the analyses.

**Table S2A:** Descriptive statistics of the categorical functional traits, showing the number of occurrences (N), the threatened and non-threatened species counts per attribute considered and a short explanation as to why these traits were grouped as such.

| Trait | Attributes | Non-threatened | Threatened | Explanation of grouping |
|---|---|---|---|---|
| Plant sex (N=707) | Dioicous | 364 | 73 | This sex clustering is widely used (Patiño and Vanderpoorten, 2018). |
| | Monoicous | 199 | 71 | |
| Capsule shape (N=580) | Cylindric | 215 | 43 | Main attributes were considered to be cylindric (shapes elongated horizontally) and globose (shapes as wide as long) after taxonomic descriptions to test their effect on spore release. |
| | Globose | 252 | 70 | |
| Capsule on seta (N=585) | Exserted | 453 | 93 | The position of the capsule held on seta is suspected to influence the spore release threshold. Grouping was applied to test this hypothesis. |
| | Immersed | 21 | 18 | |
| Seta shape (N=463) | Straight | 263 | 78 | In addition to role of seta length in spore release, this trait was grouped as such to test the effect of its shape. |
| | Curved | 108 | 14 | |
| Spore shape (N=408) | Edged | 38 | 5 | Attributes were grouped as such to test whether the aerodynamics of spore shape has an effect on the spore dispersal. |
| | Rounded | 305 | 60 | |
| Spore ornamentation (N=480) | High ornamentation | 159 | 36 | Different ornamentations were grouped as lowly/highly ornamented, inspired by Zanatta *et al.* (2016). |
| | Low ornamentation | 239 | 46 | |

**Table S2B:** Descriptive statistics of the continuous functional traits, showing the mean, standard deviation (SD), number of occurrences (N), minimum (Min) and maximum (Max) values.

| | | Non-threatened | | | Threatened | | | Total | | | | |
|---|---|---|---|---|---|---|---|---|---|---|---|---|
| **Trait** | **Unit** | **Mean** | **SD** | **N** | **Mean** | **SD** | **N** | **Mean** | **SD** | **N** | **Min** | **Max** |
| Stem length | mm | 41.43 | 51.44 | 485 | 31.08 | 36.07 | 131 | 39.23 | 48.74 | 616 | 0.55 | 600 |
| Leaf length | cm | 0.28 | 0.20 | 531 | 0.24 | 0.19 | 149 | 0.27 | 0.20 | 680 | 0.02 | 1.5 |
| Capsule length | mm | 2.02 | 1.00 | 416 | 1.50 | 0.96 | 99 | 1.92 | 1.01 | 515 | 0.2 | 8 |
| Seta length | mm | 15.33 | 13.45 | 438 | 8.30 | 11.02 | 114 | 13.88 | 13.29 | 552 | 0.0 | 130 |
| Spore diameter | $\mu$m | 19.78 | 14.93 | 466 | 20.51 | 9.36 | 89 | 19.89 | 14.18 | 555 | 6.5 | 190 |
| Substrate breadth | N | 1.78 | 0.99 | 567 | 1.24 | 0.50 | 148 | 1.67 | 0.94 | 715 | 1 | 6 |

**Table S2C:** Descriptive statistics of the binary functional traits, showing the number of occurrences per state (present or absent) (N) and per state for threatened and non-threatened species.

| **Trait** | **State** | **N** | **Non-threatened** | **Threatened** |
|---|---|---|---|---|
| Sporophyte presence | present | 662 | 530 | 132 |
| | absent | 77 | 37 | 40 |
| Vegetative reproduction | present | 109 | 97 | 12 |
| | absent | 630 | 470 | 160 |
| Persistent protonema | present | 15 | 14 | 1 |
| | absent | 724 | 553 | 171 |
