# Supplementary material for: Predicting the Threat Status of Mosses Using Functional Traits
Source: Plants (Basel). 2024 Jul 23;13(15):2019. doi: 10.3390/plants13152019 (PMC11314510; doi:10.3390/plants13152019)
Supplement: Supplementary file 1 [file plants-13-02019-s001.zip › SuppMat_Tables3A-3B.pdf]

Supplementary Material Tables S3A and S3B: The summary of the stepwise forward model selection for maximal models 1 (Table S3A) and 2 (Table S3B). The most parsimonious models (MAM1 and MAM2 accordingly) are highlighted. The degrees of freedom (df), AIC, the difference in AIC (ΔAIC, compared to MAM1 or MAM2), BIC, the difference in BIC (ΔBIC, compared to MAM1 and MAM2), Akaike weight (ω, among the models presented) and the p-values (ANOVA test result compared to MAM1 and MAM2) are reported.

3A	No.	Model	df	AIC	ΔAIC	BIC	ΔBIC	ω	p-value
MAM1	-	~ Null	590	567.05	69.16	571.44	56.02	0	<0.001
	1	~ Substrate breadth	589	521.60	23.70	530.36	14.94	0	<0.001
	2	~ Substrate breadth + Sporophyte presence	588	500.30	2.41	513.45	-1.97	0.15	0.036
	**3**	**~ Substrate breadth + Sporophyte presence + Plant sex**	**587**	**497.90**	**0**	**515.42**	**0**	**0.48**	**1**
	4	~ Substrate breadth + Sporophyte presence + Plant sex + Stem growth	586	498.43	0.54	520.34	4.92	0.37	0.226
	N=591 (109 threatened, 482 non-threatened)								

3B	No.	Model	df	AIC	ΔAIC	BIC	ΔBIC	ω	p-value
MAM2	-	~Null	426	376.42	55.55	380.47	47.43	0	<0.001
	1	~ Substrate breadth	425	331.33	10.46	339.44	6.04	0	<0.001
	**2**	**~ Substrate breadth + Seta length**	**424**	**320.87**	**0**	**333.04**	**0**	**0.45**	**1**
	3	~ Substrate breadth + Seta length + Capsule length	423	321.76	0.88	337.98	4.94	0.29	0.29
	4	~ Substrate breadth + Seta length + Capsule length + Capsule on seta	422	322.98	2.11	343.27	10.23	0.16	0.39
	5	~ Substrate breadth + Seta length + Capsule length + Capsule on seta + Plant sex	421	324.69	3.82	349.03	15.99	0.07	0.54
	6	~ Substrate breadth + Seta length + Capsule length + Capsule on seta + Plant sex + Stem length	420	326.67	5.80	355.07	22.03	0.03	0.70
	N=427 (68 threatened, 359 non-threatened)								
